# A generalised random encounter model for estimating animal density with remote sensor data

**DOI:** 10.1111/2041-210X.12346

**Published:** 2015-02-25

**Authors:** Tim C. D. Lucas, Elizabeth A. Moorcroft, Robin Freeman, J. Marcus Rowcliffe, Kate E. Jones

**Affiliations:** ^1^ CoMPLEXUniversity College LondonPhysics Building, Gower StreetLondonWC1E 6BTUK; ^2^ Centre for Biodiversity and Environment ResearchDepartment of Genetics, Evolution and EnvironmentUniversity College LondonGower StreetLondonWC1E 6BTUK; ^3^ Department of Statistical ScienceUniversity College LondonGower StreetLondonWC1E 6BTUK; ^4^ Department of Computer ScienceUniversity College LondonGower StreetLondonWC1E 6BTUK; ^5^ Institute of ZoologyZoological Society of LondonRegents ParkLondonNW1 4RYUK

**Keywords:** acoustic detection, camera traps, marine, population monitoring, simulations, terrestrial

## Abstract

1. Wildlife monitoring technology is advancing rapidly and the use of remote sensors such as camera traps and acoustic detectors is becoming common in both the terrestrial and marine environments. Current methods to estimate abundance or density require individual recognition of animals or knowing the distance of the animal from the sensor, which is often difficult. A method without these requirements, the random encounter model (REM), has been successfully applied to estimate animal densities from count data generated from camera traps. However, count data from acoustic detectors do not fit the assumptions of the REM due to the directionality of animal signals.
2. We developed a generalised REM (gREM), to estimate absolute animal density from count data from both camera traps and acoustic detectors. We derived the gREM for different combinations of sensor detection widths and animal signal widths (a measure of directionality). We tested the accuracy and precision of this model using simulations of different combinations of sensor detection widths and animal signal widths, number of captures and models of animal movement.
3. We find that the gREM produces accurate estimates of absolute animal density for all combinations of sensor detection widths and animal signal widths. However, larger sensor detection and animal signal widths were found to be more precise. While the model is accurate for all capture efforts tested, the precision of the estimate increases with the number of captures. We found no effect of different animal movement models on the accuracy and precision of the gREM.
4. We conclude that the gREM provides an effective method to estimate absolute animal densities from remote sensor count data over a range of sensor and animal signal widths. The gREM is applicable for count data obtained in both marine and terrestrial environments, visually or acoustically (e.g. big cats, sharks, birds, echolocating bats and cetaceans). As sensors such as camera traps and acoustic detectors become more ubiquitous, the gREM will be increasingly useful for monitoring unmarked animal populations across broad spatial, temporal and taxonomic scales.

## Introduction

The density of animal populations is one of the fundamental measures in ecology and conservation and has important implications for a range of issues, such as sensitivity to stochastic fluctuations (Wright & Hubbell 1983) and extinction risk (Purvis *et al*. 2000). Monitoring animal population changes in response to anthropogenic pressure is becoming increasingly important as humans rapidly modify habitats and change climates (Everatt, Andresen & Somers 2014). Sensor technology, such as camera traps (Karanth 1995; Rowcliffe & Carbone 2008) and acoustic detectors (Acevedo & Villanueva‐Rivera 2006; Walters *et al*. 2012), is widely used to monitor changes in animal populations as these sensors are efficient, relativity cheap and non‐invasive, allowing for surveys over large areas and long periods (Rowcliffe & Carbone 2008; Walters *et al*. 2013; Kessel *et al*. 2014). However, converting sampled count data into estimates of density is problematic as detectability of animals needs to be accounted for (Anderson 2001).

Existing methods for estimating animal density often require additional information that is often unavailable. For example, capture‐mark‐recapture methods (Karanth 1995; Borchers *et al*. 2014) require recognition of individuals, and distance methods (Harris *et al*. 2013) require estimates of how far away individuals are from the sensor (Barlow & Taylor 2005; Marques *et al*. 2011). When individuals cannot be told apart, an extension of occupancy modelling can be used to estimate absolute abundance (Royle & Nichols 2003). However, as the model is originally formulated to estimate occupancy, count information is simplified to presence–absence data. Assumptions about the distribution of individuals (e.g. a poisson distribution) must also be made (Royle & Nichols 2003) which may be a poor assumption for non‐randomly distributed species. Furthermore repeat, independent surveys must be performed and the definition of a site can be difficult, especially for wide‐ranging species (MacKenzie & Royle 2005).

More recently, the development of the random encounter model (REM), a modification of an ideal gas model (Yapp 1956; Hutchinson & Waser 2007), has enabled animal densities to be estimated from unmarked individuals with a known speed, and sensor detection parameters (Rowcliffe *et al*. 2008). The REM method has been successfully applied to estimate animal densities from camera trap surveys (Zero *et al*. 2013). However, extending the REM method to other types of sensors (e.g. acoustic detectors) is more problematic, because the original derivation assumes a relatively narrow sensor width (up to π/2 radians) and that the animal is equally detectable irrespective of its heading (Rowcliffe *et al*. 2008).

Whilst these restrictions are not problematic for most camera trap makes (e.g. Reconyx, Cuddeback), the REM cannot be used to estimate densities from camera traps with a wider sensor width [e.g. canopy monitoring with fish eye lenses, Brusa & Bunker (2014)]. Additionally, the REM method is not useful in estimating densities from acoustic survey data as acoustic detector angles are often wider than π/2 radians. Acoustic detectors are designed for a range of diverse tasks and environments (Kessel *et al*. 2014), which naturally leads to a wide range of sensor detection widths and detection distances. In addition to this, calls emitted by many animals are directional (Blumstein *et al*. 2011), breaking the assumption of the REM method.

There has been a sharp rise in interest around passive acoustic detectors in recent years, with a 10‐fold increase in publications in the decade between 2000 and 2010 (Kessel *et al*. 2014). Acoustic monitoring is being developed to study many aspects of ecology, including the interactions of animals and their environments (Blumstein *et al*. 2011; Rogers *et al*. 2013), the presence and relative abundances of species (Marcoux *et al*. 2011), biodiversity of an area (Depraetere *et al*. 2012) and monitoring population trends (Walters *et al*. 2013).

Acoustic data suffer from many of the problems associated with data from camera trap surveys in that individuals are often unmarked, making capture‐mark‐recapture methods more difficult to use (Marques *et al*. 2013). In some cases, the distance between the animal and the sensor is known, for example when an array of sensors is deployed and the position of the animal is estimated by triangulation (Lewis *et al*. 2007). In these situations, distance‐sampling methods can be applied (Buckland, Marsden & Green 2008). However, in many cases, distance estimation is not possible, for example when single sensors are deployed, a situation typical in the majority of terrestrial acoustic surveys (Buckland, Marsden & Green 2008). In these cases, only relative measures of local abundance can be calculated and not absolute densities. This means that comparison of populations between species and sites is problematic without assuming equal detectability (Schmidt 2003; Walters *et al*. 2013). Equal detectability is unlikely because of differences in environmental conditions, sensor type, habitat and species biology.

In this study, we create a generalised REM (gREM) as an extension to the camera trap model of Rowcliffe *et al*. (2008), to estimate absolute density from count data from acoustic detectors, or camera traps, where the sensor width can vary from 0 to 2π radians, and the signal given from the animal can be directional. We assessed the accuracy and precision of the gREM within a simulated environment, by varying the sensor detection widths, animal signal widths, number of captures and models of animal movement. We use the simulation results to recommend best survey practice for estimating animal densities from remote sensors.

## Materials and methods

### Analytical model

**Figure 1:**
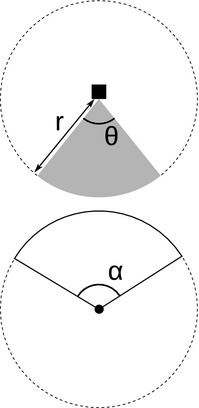
Representation of sensor detection width and animal signal width. The filled square and circle represent a sensor and an animal, respectively; θ, sensor detection width (radians); *r*, sensor detection distance; dark grey shaded area, sensor detection zone; α, animal signal width (radians). Dashed lines around the filled square, and circle represents the maximum extent of θ and α, respectively.

The REM presented by Rowcliffe *et al*. (2008) adapts the gas model to count data collected from camera trap surveys. The REM is derived assuming a stationary sensor with a detection width < π/2 radians. However, in order to apply this approach more generally, and in particular to stationary acoustic detectors, we need both to relax the constraint on sensor detection width and allow for animals with directional signals. Consequently, we derive the gREM for any detection width, θ, between 0 and 2π with a detection distance *r* giving a circular sector within which animals can be captured (the detection zone) (Fig. 1). Additionally, we model the animal as having an associated signal width α between 0 and 2π (Fig. 1, see Appendix S1 for a list of symbols). We start deriving the gREM with the simplest situation, the gas model where θ = 2π and α = 2π.

#### Gas model

Following Yapp (1956), we derive the gas model where sensors can capture animals in any direction and animal signals are detectable from any direction (θ = 2π and α = 2π). We assume that animals are in a homogeneous environment and move in straight lines of random direction with velocity *v*. We allow that our stationary sensor can capture animals at a detection distance *r* and that if an animal moves within this detection zone they are captured with a probability of one; while outside this zone, animals are never captured.

In order to derive animal density, we need to consider relative velocity from the reference frame of the animals. Conceptually, this requires us to imagine that all animals are stationary and randomly distributed in space, while the sensor moves with velocity *v*. If we calculate the area covered by the sensor during the survey period, we can estimate the number of animals the sensor should capture. As a circle moving across a plane, the area covered by the sensor per unit time is 2*rv*. The expected number of captures, *z*, for a survey period of *t*, with an animal density of *D* is *z* = 2*rvtD*. To estimate the density, we rearrange to get *D* = *z*/2*rvt*. Note that as *z* is the number of encounters, not individuals, the possibility of repeated detections of the same individual is accounted for (Hutchinson & Waser 2007).

#### gREM derivations for different detection and signal widths

Different combinations of θ and α would be expected to occur (e.g. sensors have different detection widths and animals have different signal widths). For different combinations θ and α, the area covered per unit time is no longer given by 2*rv*. Instead of the size of the sensor detection zone having a diameter of 2*r*, the size changes with the approach angle between the sensor and the animal. The width of the area within which an animal can be detected is called the profile, *p*. The size of *p* depends on the signal width, detector width and the angle that the animal approaches the sensor. The size of the profile (averaged across all approach angles) is defined as the average profile $\overset{¯}{p}$. However, different combinations of θ and α need different equations to calculate $\overset{¯}{p}$.

**Figure 2:**
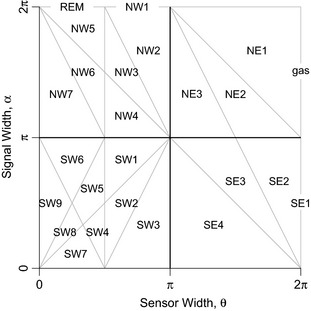
Locations where derivation of the average profile $\overset{¯}{p}$ is the same for different combinations of sensor detection and animal signal widths. Symbols within each polygon refer to each gREM submodel named after their compass point, except for Gas and REM which highlight the position of these previously derived models within the gREM. Symbols on the edge of the plot are for submodels where α, θ = 2π.

We have identified the parameter space for the combinations of θ and α for which the derivation of the equations is the same (defined as submodels in the gREM) (Fig. 2). For example, the gas model becomes the simplest gREM submodel (upper right in Fig. 2), and the REM from Rowcliffe *et al*. (2008) is another gREM submodel where θ < π/2 and α = 2π. We derive one gREM submodel SE2 as an example below, where 2π−α/2 < θ < 2π, 0 < α < π (see Appendix S2 for derivations of all gREM submodels). Any estimate of density would require prior knowledge of animal velocity, *v* and animal signal width, α taken from other sources, for example existing literature (Carbone *et al*. 2005; Brinklov *et al*. 2011). Sensor width, θ, and detection distance, *r* would also need to be measured or obtained from manufacturer specifications (Holderied & Von Helversen 2003; Adams *et al*. 2012).

#### Example derivation of SE2

**Figure 3:**
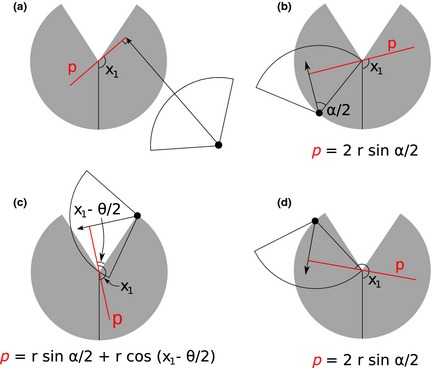
An overview of the derivation of the average profile $\overset{¯}{p}$ for the gREM submodel SE2, where (a) shows the location of the profile *p* (the line an animal must pass through in order to be captured) in red and the focal angle, $x_{1}$, for an animal (filled circle), its signal (unfilled sector), and direction of movement (shown as an arrow). The detection zone of the sensor is shown as a filled grey sector with a detection distance of *r*. The vertical black line within the circle shows the direction the sensor is facing. The derivation of *p* changes as the animal approaches the sensor from different directions (shown in b–d), where (b) is the derivation of *p* when $x_{1}$ is in the interval [π/2,π/2+θ/2−α/2], (c) *p* when $x_{1}$ is in the interval [π/2+θ/2−α/2,5π/2−θ/2−α/2] and (d) *p* when $x_{1}$ is in the interval [5π/2−θ/2−α/2,3π/2], where θ, sensor detection width; α, animal signal width. The resultant equation for *p* is shown beneath b–d. The average profile $\overset{¯}{p}$ is the size of the profile averaged across all approach angles.

In order to calculate $\overset{¯}{p}$, we have to integrate over the focal angle, $x_{1}$ (Fig. 3a). This is the angle taken from the centre line of the sensor. Other focal angles are possible ($x_{2}$, $x_{3}$, $x_{4}$) and are used in other gREM submodels (see Appendix S2). As the size of the profile depends on the approach angle, we present the derivation across all approach angles. When the sensor is directly approaching the animal $x_{1}=\pi /2$.

Starting from $x_{1}=\pi /2$ until θ/2+π/2−α/2, the size of the profile is 2*r* sin α/2 (Fig. 3b). During this first interval, the size of α limits the width of the profile. When the animal reaches $x_{1}$ = θ/2+π/2−α/2 (Fig. 3c), the size of the profile is $r\sin\left(\alpha /2\right)+r\cos\left(x_{1}-\theta /2\right)$, and the size of θ and α both limit the width of the profile (Fig. 3c). Finally, at $x_{1}=5\pi /2-\theta /2-\alpha /2$ until $x_{1}=3\pi /2$, the width of the profile is again 2*r* sin α/2 (Fig. 3d) and the size of α again limits the width of the profile.

The profile width *p* for π radians of rotation (from directly towards the sensor to directly behind the sensor) is completely characterised by the three intervals (Fig. 3b–d). Average profile width $\overset{¯}{p}$ is calculated by integrating these profiles over their appropriate intervals of $x_{1}$ and dividing by π which gives

(eqn 1) $$\hat{p}=\frac{1}{\pi }\left(\int_{\frac{\pi }{2}}^{\frac{\pi }{2}+\frac{\theta }{2}-\frac{\alpha }{2}}2r\sin\frac{\alpha }{2}\;dx_{1}+\int_{\frac{\pi }{2}+\frac{\theta }{2}-\frac{\alpha }{2}}^{\frac{5\pi }{2}-\frac{\theta }{2}-\frac{\alpha }{2}}r\sin\frac{\alpha }{2}+r\cos\left(x_{1}-\frac{\theta }{2}\right)\;dx_{1}+\int_{\frac{5\pi }{2}-\frac{\theta }{2}-\frac{\alpha }{2}}^{\frac{3\pi }{2}}2r\sin\frac{\alpha }{2}\;dx_{1}\right)$$

(eqn 2) $$=\frac{r}{\pi }\left(\theta \sin\frac{\alpha }{2}-\cos\frac{\alpha }{2}+\cos\left(\frac{\alpha }{2}+\theta \right)\right)$$

We then use this expression to calculate density

(eqn 3) $$D=z/vt\overset{¯}{p}.$$

Rather than having one equation that describes $\overset{¯}{p}$ globally, the gREM must be split into submodels due to discontinuous changes in *p* as α and β change. These discontinuities can occur for a number of reasons such as a profile switching between being limited by α and θ, the difference between very small profiles and profiles of size zero, and the fact that the width of a sector stops increasing once the central angle reaches π radians (i.e. a semi‐circle is just as wide as a full circle). As an example, if α is small, there is an interval between Fig. 3c,d where the ‘blind spot’ would prevent animals being detected giving *p* = 0. This would require an extra integral in our equation, as simply putting our small value of α into 1 would not give us this integral of *p* = 0.

gREM submodel specifications were done by hand, and the integration was done using SymPy (SymPy Development Team 2014) in Python (Appendix S3). The gREM submodels were checked by confirming that: (i) submodels adjacent in parameter space were equal at the boundary between them; (ii) submodels that border α=0 had *p*=0 when α=0; (iii) average profile widths $\overset{¯}{p}$ were between 0 and 2*r* and; (iv) each integral, divided by the range of angles that it was integrated over, was between 0 and 2*r*. The scripts for these tests are included in Appendix S3, and the R (R Core Team 2014) implementation of the gREM is given in Appendix S4.

### Simulation model

We tested the accuracy and precision of the gREM by developing a spatially explicit simulation of the interaction of sensors and animals using different combinations of sensor detection widths, animal signal widths, number of captures and models of animal movement. One hundred simulations were run where each consisted of a 7·5 km by 7·5 km square with periodic boundaries. A stationary sensor of radius *r*, 10 m, was set up in the exact centre of each simulated study area, covering seven sensor detection widths θ, between 0 and 2π (2/9π, 4/9π, 6/9π, 8/9π, 10/9π, 14/9π, and 2π). Each sensor was set to record continuously and to capture animal signals instantaneously from emission. Each simulation was populated with a density of 70 animals km^−2^, calculated from the equation in Damuth (1981) as the expected density of mammals weighing 1 g. This density therefore represents a reasonable upper estimate of density of individuals, given that the smallest mammal is around 2 g (Jones *et al*. 2009). A total of 3937 individuals per simulation were created which were placed randomly at the start of the simulation. A total of 11 signal widths α between 0 and π were used (1/11π, 2/11π, 3/11π, 4/11π, 5/11π, 6/11π, 7/11π, 8/11π, 9/11π, 10/11π, π).

Each simulation lasted for *N* steps (14 400) of duration *T* (15 min) giving a total duration of 150 days. The individuals moved within each step with a distance *d*, with an average speed, *v*. The distance, *d*, was sampled from a normal distribution with mean distance, $\mu_{d}=vT$, and standard deviation, $\sigma_{d}=vT/10$, where the standard deviation was chosen to scale with the average distance travelled. An average speed, *v*= 40  km day^−1^, was chosen based on the largest day range of terrestrial animals (Carbone *et al*. 2005) and represents the upper limit of realistic speeds. At the end of each step, individuals were allowed to either remain stationary for a time step (with a given probability, *S*) or change direction where the change in direction has a uniform distribution in the interval [−*A*, *A*]. This resulted in seven different movement models where: (1) simple movement, where *S* and *A* = 0; (2) stop‐start movement, where (i) *S* = 0·25, *A* = 0, (ii) *S* = 0·5, *A* = 0, (iii) *S* = 0·75, *A* = 0; (3) correlated random walk movement, where (i) *S* = 0, *A* = π/3, (ii) *S* = 0, *A* = 2π/3, (iii) *S* = 0, *A* = π. Encounters per simulation were counted as they moved into the detection zone of the sensor.

We calculated the estimated animal density from the gREM by summing the number of captures per simulation and inputting these values into the correct gREM submodel. The accuracy of the gREM was determined by comparing the true simulation density with the estimated density. Precision of the gREM was determined by the standard deviation of estimated densities. We used this method to compare the accuracy and precision of all the gREM submodels. As these submodels are derived for different combinations of α and θ, the accuracy and precision of the submodels were used to determine the impact of different values of α and θ.

The influence of the number of captures and animal movement models on accuracy and precision was investigated using four different gREM submodels representative of the range α and θ values (submodels NW1, SW1, NE1, and SE3, Fig. 2). From a random starting point, we ran the simulation until a range of different capture numbers were recorded (from 10 to 100 captures), recorded the length of time this took and estimated the animal density for each of the four submodels. These estimated densities were compared to the true density to assess the impact on the accuracy and precision of the gREM. We calculated the coefficient of variation in order to compare the precision of the density estimates from simulations with different expected numbers of captures. The gREM also assumes that individuals move continuously with straight‐line movement (simple movement model) and we therefore assessed the impact of breaking the gREM assumptions. We used the four submodels to compare the accuracy and precision of a simple movement model, stop–start movement models (using different average amounts of time spent stationary) and random walk movement models. Finally, as the parameters (α, β, *r* and *v*) are likely to be measured with error, we compared true simulation densities to densities estimated with parameters with errors of 0%, ±5% and ±10%, for all gREM submodels.

## Results

### Analytical model

**Figure 4:**
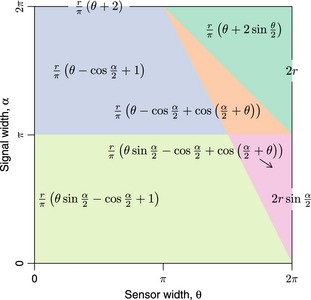
Expressions for the average profile width,$\;\;\overset{¯}{p}$ given a range of sensor and signal widths. Despite independent derivation within each block, many models result in the same expression. These are collected together and presented as one block of colour. Expressions on the edge of the plot are for submodels with α, θ = 2*π*.

The equation for $\overset{¯}{p}$ has been newly derived for each submodel in the gREM, except for the gas model and REM which have been calculated previously. However, many models, although derived separately, have the same expression for $\overset{¯}{p}$. Figure 4 shows the expression for $\overset{¯}{p}$ in each case. The general equation for density, eqn 3, is used with the correct value of $\overset{¯}{p}$ substituted. Although more thorough checks are performed in Appendix S3, it can be seen that all adjacent expressions in Fig. 4 are equal when expressions for the boundaries between them are substituted in.

### Simulation model

#### gREM submodels

**Figure 5:**
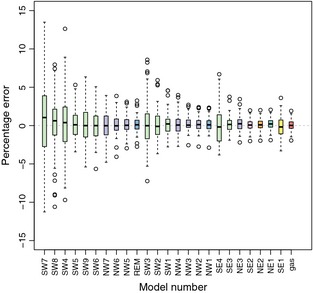
Simulation model results of the accuracy and precision for gREM submodels. The percentage error between estimated and true density for each gREM submodel is shown within each box plot, where the black line represents the median percentage error across all simulations, boxes represent the middle 50% of the data, whiskers represent variability outside the upper and lower quartiles with outliers plotted as individual points. Box colours correspond to the expressions for average profile width $\overset{¯}{p}$ given in Figure 4.

All gREM submodels showed a high accuracy, that is the median difference between the estimated and true values was < 2% across all models (Fig. 5). However, the precisions of the submodels do vary, where the gas model is the most precise and the SW7 submodel the least precise, having the smallest and the largest interquartile range, respectively (Fig. 5). The standard deviation of the error between the estimated and true densities is strongly related to both the sensor and signal widths (Appendix S5), such that larger widths have lower standard deviations (greater precision) due to the increased capture rate of these models.

#### Number of captures

**Figure 6:**
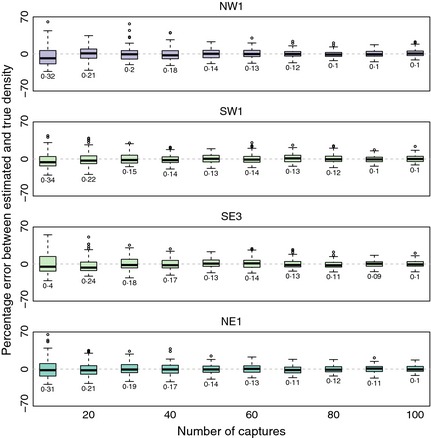
Simulation model results of the accuracy and precision of four gREM submodels (NW1, SW1, SE3 and NE1) given different numbers of captures. The percentage error between estimated and true density within each gREM submodel for capture rate is shown within each box plot, where the black line represents the median percentage error across all simulations, boxes represent the middle 50% of the data, whiskers represent variability outside the upper and lower quartiles with outliers plotted as individual points. Sensor and signal widths vary between submodels. The numbers beneath each plot represent the coefficient of variation. The colour of each box plot corresponds to the expressions for average profile width $\overset{¯}{p}$ given in Figure 4.

Within the four gREM submodels tested (NW1, SW1, SE3, NE1), the accuracy was not strongly affected by the number of captures. The median difference between the estimated and true values was < 15% across all capture rates (Fig. 6). However, the precision was dependent on the number of captures across all four of the gREM submodels, where precision increases as number of captures increases, as would be expected for any statistical estimate (Fig. 6). For all gREM submodels, the coefficient of variation falls to 10% at 100 captures.

#### Movement models

**Figure 7:**
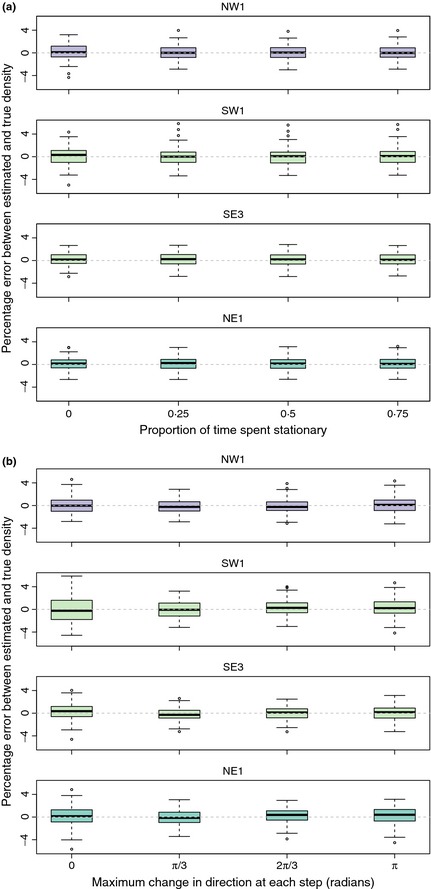
Simulation model results of the accuracy and precision of four gREM submodels (NW1, SW1, SE3 and NE1) given different movement models where (a) average amount of time spent stationary (stop‐start movement) and (b) maximum change in direction at each step (correlated random walk model). The percentage error between estimated and true density within each gREM submodel for the different movement models is shown within each box plot, where the black line represents the median percentage error across all simulations, boxes represent the middle 50% of the data, whiskers represent variability outside the upper and lower quartiles with outliers plotted as individual points. The simple model is represented where time and maximum change in direction equals 0. The colour of each box plot corresponds to the expressions for average profile width $\overset{¯}{p}$ given in Figure 4.

Within the four gREM submodels tested (NW1, SW1, SE3, NE1), neither the accuracy nor precision was affected by the average amount of time spent stationary. The median difference between the estimated and true values was < 2% for each category of stationary time (0, 0·25, 0·5 and 0·75) (Fig. 7a). Altering the maximum change in direction in each step (0, π/3, 2π/3, and π) did not affect the accuracy or precision of the four gREM submodels (Fig. 7b).

#### Impact of parameter error

The percentage error in the density estimates across all parameters, and gREM submodels shows a similar response for under and over estimated parameters, suggesting the accuracy is reasonable with respect to parameter error (Appendix S6). The impact of parameter error on the precision of the density estimate varies across gREM submodels and parameters, where α shows the largest variation including the largest values. However, in all cases, the percentage error in the density estimate is not more than 5% greater than the error in the parameter estimate (Appendix S6).

## Discussion

### Analytical model

We have developed the gREM such that it can be used to estimate density from acoustic sensors and camera traps. This has entailed a generalisation of the gas model and the REM in Rowcliffe *et al*. (2008) to be applicable to any combination of sensor width θ and signal directionality α. We emphasise that the approach is robust to multiple detections of the same individual. We have used simulations to show, as a proof of principle, that these models are accurate and precise.

There are a number of possible extensions to the gREM that could be developed in the future. The original gas model was formulated for the case where both animals and sensors are moving (Hutchinson & Waser 2007). Indeed, any of the models which have animals that are equally detectable in all directions (α = 2π) can be trivially expanded by replacing animal speed *v* with $v+v_{s}$ where $v_{s}$ is the speed of the sensor. However, when the animal has a directional call the extension becomes less simple. The approach would be to calculate again the mean profile width. However, for each angle of approach, one would have to average the profile width for an animal facing in any direction (i.e. not necessarily moving towards the sensor) weighted by the relative velocity of that direction. There are a number of situations where a moving detector and animal could occur, for example an acoustic detector towed from a boat when studying porpoises (Kimura *et al*. 2014) or surveying echolocating bats from a moving car (Jones *et al*. 2013).

Interesting but unstudied problems impacting the gREM are firstly, edge effects caused by sensor trigger delays (the delay between sensing an animal and attempting to record the encounter) (Rovero *et al*. 2013), and secondly, sensors which repeatedly turn on an off during sampling (Jones *et al*. 2013). The second problem is particularly relevant to acoustic detectors which record ultrasound by time expansion. Here, ultrasound is recorded for a set time period and then slowed down and played back, rendering the sensor ‘deaf’ periodically during sampling. Both of these problems may cause biases in the gREM, as animals can move through the detection zone without being detected. As the gREM assumes constant surveillance, the error created by switching the sensor on and off quickly will become more important if the sensor is only on for short periods of time. We recommend that the gREM is applied to constantly sampled data, and the impacts of breaking these assumptions on the gREM should be further explored.

### Accuracy, precision and recommendations for best practice

Based on our simulations, we believe that the gREM has the potential to produce accurate estimates for many different species, using either camera traps or acoustic detectors. However, the precision of the gREM differed between submodels. For example, when the sensor and signal width were small, the precision of the model was reduced. Therefore, when choosing a sensor for use in a gREM study, the sensor detection width should be maximised. If the study species has a narrow signal directionality, other aspects of the study protocol, such as length of the survey, should be used to compensate.

The precision of the gREM is greatly affected by the number of captures. The coefficient of variation falls dramatically between 10 and 60 captures and then after this continues to slowly reduce. At 100 captures, the submodels reach 10% coefficient of variation, considered to be a very good level of precision and better than many previous studies (O'Brien, Kinnaird & Wibisono 2003; Foster & Harmsen 2012; Thomas & Marques 2012). The length of surveys in the field will need to be adjusted so that enough data can be collected to reach this precision level. Populations of fast moving animals or populations with high densities will require less survey effort than those species that are slow moving or have populations with low densities.

We found that the sensitivity of the gREM to inaccurate parameter estimates was both predictable and reasonable (Appendix S6), although this varies between different parameters and gREM submodels. Whilst care should be taken in parameter estimation when analysing both acoustic and camera trap data, acoustic data pose particular problems. For acoustic surveys, estimates of *r* (detection distance) can be measured directly or calculated using sound attenuation models (Holderied & Von Helversen 2003), while the sensor angle is often easily measured (Adams *et al*. 2012) or found in the manufacturer's specifications. When estimating animal movement speed *v*, only the speed of movement during the survey period should be used. The signal width is the most sensitive parameter to inaccurate estimates (Appendix S6) and is also the most difficult to measure. While this parameter will typically be assumed to be 2π for camera trap surveys, fewer estimates exist for acoustic signal widths. Although signal width has been measured for echolocating bats using arrays of microphones (Brinklov *et al*. 2011), more work should be done on obtaining estimates for a range of acoustically surveyed species.

### Limitations

Although the REM has been found to be effective in field tests (Rowcliffe *et al*. 2008; Zero *et al*. 2013), the gREM requires further validation by both field tests and simulations. For example, capture‐mark‐recapture methods could be used alongside the gREM to test the accuracy under field conditions (Rowcliffe *et al*. 2008). While we found no effect of the movement model on the accuracy or precision of the gREM, the models we have used in our simulations to validate the gREM are still simple representations of true animal movement. Animal movement may be highly nonlinear and often dependent on multiple factors such as behavioural state and existence of home ranges (Smouse *et al*. 2010). Therefore, testing the gREM against real animal data, or further simulations with more complex movement models, would be beneficial.

The assumptions of our simulations may require further consideration, for example we have assumed an equal density across the study area. However, in a field environment, the situation may be more complex, with additional variation coming from local changes in density between sensor sites. Although unequal densities should theoretically not affect accuracy (Hutchinson & Waser 2007), it will affect precision and further simulations should be used to quantify this effect. Additionally, we allowed the sensor to be stationary and continuously detecting, negating the triggering, and non‐continuous recording issues that could exist with some sensors and reduce precision or accuracy. Finally, in the simulation animals moved at the equivalent of the largest day range of terrestrial animals (Carbone *et al*. 2005). Slower speed values should not alter the accuracy of the gREM, but precision would be affected since slower speeds produce fewer records.

A feature of the gREM is that it does not fit a statistical model to estimate detection probability as occupancy models and distance sampling do (Royle & Nichols 2003; Barlow & Taylor 2005; Marques *et al*. 2011). Instead, it explicitly models the process, with animals only being detected if they approach the sensor from a suitable direction. Other processes that affect detection probability could be included in the model to improve realism.

### Implications for ecology and conservation

The gREM is applicable for count data obtained either visually or acoustically in both marine and terrestrial environments and is suitable for taxa including echolocating bats (Walters *et al*. 2012), songbirds (Buckland & Handel 2006), whales (Marques *et al*. 2011) and forest primates (Hassel‐Finnegan *et al*. 2008). Many of these taxa contain critically endangered species, and monitoring their populations is of conservation interest. For example, current methods of density estimation for the threatened Franciscana dolphin (*Pontoporia blainvillei*) may result in underestimation of their numbers (Crespo *et al*. 2010). In addition, using gREM may be easier than other methods for measuring the density of animals which may be useful in quantifying ecosystem services, such as songbirds with a known positive influence on pest control (Jirinec, Campos & Johnson 2011).

The gREM will aid researchers to study species with non‐invasive methods such as remote sensors, which allows for large, continuous monitoring projects with limited human resources (Kelly *et al*. 2012). The gREM is also suitable for species that are sensitive to human contact or are difficult or dangerous to catch (Thomas & Marques 2012). As sensors such as camera traps and acoustic detectors become more ubiquitous, the gREM will be increasingly useful for monitoring unmarked animal populations across broad spatial, temporal and taxonomic scales.

## Supporting information


**Appendix S1–S6.** Supplementary Figures and Tables (Table S1, Figures S1–S6).
