# Supplementary material for: A generalised random encounter model for estimating animal density with remote sensor data
Source: Methods Ecol Evol. 2015 Feb 25;6(5):500–9. doi: 10.1111/2041-210X.12346 (PMC4974944; doi:10.1111/2041-210X.12346)
Supplement: Supplementary file 1 — Appendix S1–S6. Supplementary Figures and Tables (Table S1, Figures S1–S6). [file MEE3-6-500-s001.pdf]

# SUPPLEMENTARY INFORMATION: A GENERALISED RANDOM ENCOUNTER MODEL FOR ESTIMATING ANIMAL DENSITY WITH REMOTE SENSOR DATA

**Authors:**
Tim C.D. Lucas[1,2,3]†, Elizabeth A. Moorcroft[1,4,5]†, Robin Freeman[5], Marcus J. Rowcliffe[5], Kate E. Jones[2,5]

**Addresses:**
1 CoMPLEX, University College London, Physics Building, Gower Street, London, WC1E 6BT, UK
2 Centre for Biodiversity and Environment Research, Department of Genetics, Evolution and Environment, University College London, Gower Street, London, WC1E 6BT, UK
3 Department of Statistical Science, University College London, Gower Street, London, WC1E 6BT, UK
4 Department of Computer Science, University College London, Gower Street, London, WC1E 6BT, UK
5 Institute of Zoology, Zoological Society of London, Regents Park, London, NW1 4RY, UK
† First authorship shared.

## S1. Table of symbols

| Symbol | Description | Units |
|---|---|---|
| $\theta$ | Sensor width | rad |
| $\alpha$ | Animal signal width | rad |
| $x_i$ | Focal angle, $i \in \{1, 2, 3, 4\}$ | rad |
| $r$ | Detection distance | m |
| $\bar{p}$ | Average profile width | m |
| $p$ | A specific profile width | m |
| $v$ | Velocity | $\mathrm{m\,s^{-1}}$ |
| $t$ | Time | s |
| $z$ | Number of detections | - |
| $D$ | Animal density | $\mathrm{m^{-2}}$ |
| $T$ | Step length | s |
| $N$ | Number of steps per simulation | - |
| $d$ | Distance moved in a time step | m |
| $S$ | Probability of remaining stationary | - |
| $A$ | Maximum turning angle | rad |

Table S1. List of symbols used to describe the gREM and simulations. '-' means the quantity has no units.

## S2. Supplementary Methods

S2.1. **Introduction.** These supplementary methods derive all the models used. For continuity, the gas model derivation is included here as well as in the main text. The calculation of all integrals use in the gREM is included in the Python script S3.

S2.2. **Gas model.** Following **?**, we derive the gas model where sensors can capture animals in any direction and animal signals are detectable from any direction ($\theta = 2\pi$ and $\alpha = 2\pi$). We assume that animals are in a homogeneous environment, and move in straight lines of random direction with velocity $v$. We allow that our stationary sensor can capture animals at a detection distance $r$ and that if an animal moves within this detection zone they are captured with a probability of one, while animals outside the zone are never captured.

In order to derive animal density, we need to consider relative velocity from the reference frame of the animals. Conceptually, this requires us to imagine that all animals are stationary and randomly distributed in space, while the sensor moves with velocity $v$. If we calculate the area covered by the sensor during the survey period we can estimate the number of animals the sensor should capture. As a circle moving across a plane, the area covered by the sensor per unit time is $2rv$. The number of expected captures, $z$, for a survey period of $t$, with an animal density of $D$ is $z = 2rvtD$. To estimate the density, we rearrange to get $D = z/2rvt$.

S2.2.1. *gREM derivations for different detection and signal widths.* Different combinations of $\theta$ and $\alpha$ would be expected to occur (e.g., sensors have different detection widths and animals have different signal widths). For different combinations $\theta$ and $\alpha$, the area covered per unit time is no longer given by $2rv$. Instead of the size of the sensor detection zone having a diameter of $2r$, the size changes with the approach angle between the sensor and the animal. For any given signal width and detector width and depending on the angle that the animal approaches the sensor, the width of the area within which an animal can be detected is called the profile, $p$. The size of the profile (averaged across all approach angles) is defined as the average profile $\bar{p}$. However, different combinations of $\theta$ and $\alpha$ need different equations to calculate $\bar{p}$. This $\bar{p}$ is the only thing that changes

We have identified the parameter space for the combinations of $\theta$ and $\alpha$ for which the derivation of the equations are the same (defined as sub-models in the gREM) (Fig. S2.1). For example, the gas

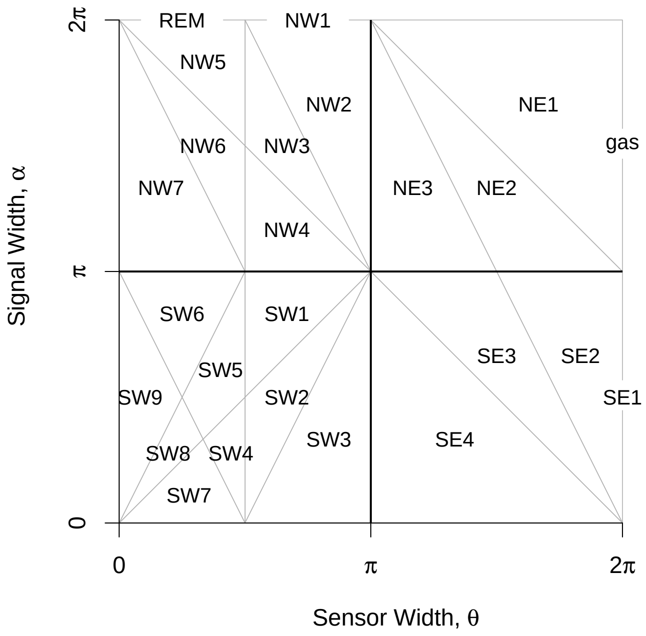


Figure S2.1. Locations where derivation of the average profile $\bar{p}$ is the same for different combinations of sensor detection and animal signal widths. Symbols within each polygon refer to each gREM submodel named after their compass point, except for Gas and REM which highlight the position of these previously derived models within the gREM. Symbols on the edge of the plot are for submodels where $\alpha, \theta = 2\pi$

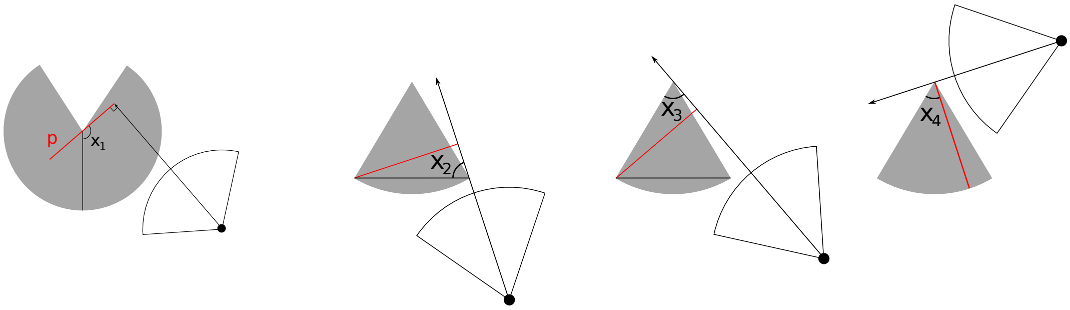


(a) $x_1$ (b) $x_2$ (c) $x_3$ (d) $x_4$

Figure S2.2. The location of the focal angles $x_{i\in[1,4]}$. $x_1$ is used in SE and NE models (including the gas model). $x_2 - x_4$ are used in NW and SW models. The sector shaped detection region is shown in grey. Animals are filled black circles and the animal signal is an unfilled sector. The animals direction of movement is indicated with an arrow. The profile $p$ is shown with a red line. (a) Animal is directly approaching the sensor at $x_1 = \frac{\pi}{2}$. (b) Animal is directly approaching the sensor at $x_2 = \frac{\pi}{2}$. $x_2$ then decreases until the profile is perpendicular to the edge of the detection region. (c) When the profile is perpendicular to the edge of the detection region, $x_3 = \theta$. (d) $x_4$ measures the angle between the left side of the detection region and the profile.

model becomes the simplest gREM sub-model (upper right in Fig. S2.1) and the REM from (**?**) is another gREM sub-model where $\theta < \pi/2$ and $\alpha = 2\pi$.

Models with $\theta = 2\pi$ are described first (the gas model described above and SE1). Then models with $\theta > \pi$ are described (NE then SE). Finally models with $\theta < \pi$ (NW then SW) are described.

S2.3. **Model SE1.** SE1 is very similar to the gas model except that because $\alpha \leq \pi$ the profile width is no longer $2r$ but is instead limited by the width of the animal signal. We therefore get a profile width of $2r\sin(\alpha/2)$ instead.

$$\bar{p}_{\text{SE1}} = \frac{1}{\pi}\int_{\frac{\pi}{2}}^{\frac{3\pi}{2}} 2r\sin\left(\frac{\alpha}{2}\right)\mathrm{d}x_1 \qquad \text{eqn S1}$$

$$\bar{p}_{\text{SE1}} = 2r\sin\left(\frac{\alpha}{2}\right) \qquad \text{eqn S2}$$

This profile is integrated over the interval $[\frac{\pi}{2}, \frac{3\pi}{2}]$ which is $\pi$ radians of rotation starting with the animal moving directly towards the sensor (Fig. S2.2a).

S2.4. **Models NE1–3.** When the detection zone is not a circle, we have more complex profiles and need to explicitly write functions for the width of the profile for every approach angle. We then use these functions to find the average profile width $\bar{p}$ for all approach angles by integrating across all $2\pi$ angles of approach and dividing by $2\pi$.

There are three submodels within quadrant NE (Fig. S2.1). Note that NE1 covers the area $\alpha = 2\pi$ as well as the triangle below it as these two models are specified exactly the same, rather than happening to have equal results.

These models have up to five profiles.

(1) The profile width starts, from $x_1 = \frac{\pi}{2}$ as $2r$.
(2) At $x_1 = \theta/2$, the right hand side of the profile cannot be $r$ wide as the corner of the 'blind spot' limits its size to being $r\cos(x_1 - \theta/2)$ wide (Fig. S2.3a).
(3) The third profile is only found in NE3. If $\alpha < 4\pi - 2\theta$, then at $x_1 = \theta/2 + \pi/2$, when the profile is perpendicular to the edge of the blind spot, the whole right side of the profile is invisible to the sensor (Fig. S2.3b). This gives a profile size of just $r$.
(4) At some point, the sensor can detect animals once they have passed the blind spot giving a profile width of $r + r\cos(x_1 + \theta/2)$ (Fig. S2.3c). From $x_1 = \pi$, if the animal signal is wide enough to be detected in this area, this is the wider profile. This then defines the split between NE1

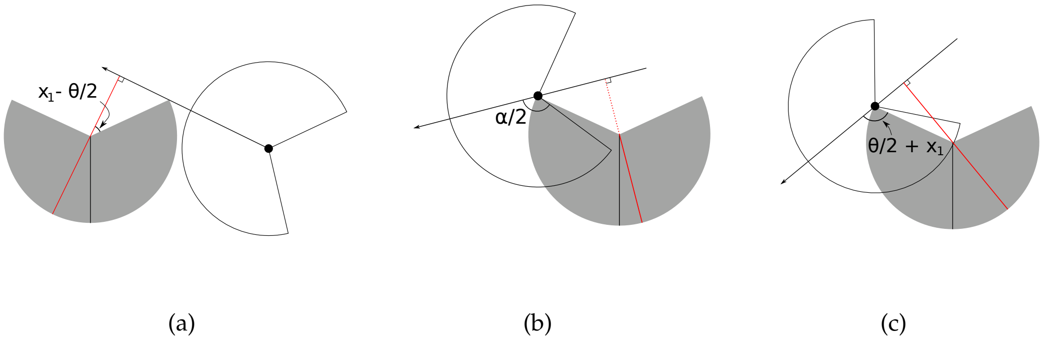


Figure S2.3. Three of the integrals in NE models. The sector shaped detection region is shown in grey. Animals are filled black circles and the animal signal is an unfilled sector. The animals direction of movement is indicated with an arrow. The profile $p$ is shown with a red line. Dashed red lines indicate areas where animals cannot be detected. (a) The second integral in NE with width $r + r\cos(x_1 - \theta/2)$ (b) The third integral in NE3. $\alpha/2$ is labelled. As it is small, animals to the right of the detector cannot be detected. (c) After further rotation, $\alpha/2$ is now bigger than the angle shown and animals to the right of the detector can again be detected.

and NE2. In NE1, with $\alpha > 3\pi - \theta$, the animal signal is wide enough that at $x_1 = \pi$ the animal can immediately be detected past the blind spot and so this profile is used. In NE2, with $\alpha < 3\pi - \theta$, the latter profile is reached at $5\pi/2 - \theta/2 - \alpha/2$.

(5) Finally, common to all three models, at $x_1 = 2\pi - \theta/2$ the profile becomes a full $2r$ once again.

S2.4.1. *Model NE1.* Submodel NE1 exists within the area bounded by $\alpha \leq 2\pi$, $\theta \leq 2\pi$ and $\alpha \geq 3\pi - \theta$ (Fig. S2.1). It has four profiles; it does not include the $r$ profile at $x_1 = \pi$ (profile described in point (3) in Section S2.4). Furthermore, $\theta$ is wide enough that the $r + r\cos(x_1 + \theta/2)$ profile starts at $\pi$. This then gives us

$$\begin{aligned}\bar{p}_{\mathrm{NE1}} =& \frac{1}{\pi}\left( \int_{\frac{\pi}{2}}^{\frac{\theta}{2}} 2r \,\mathrm{d}x_1 + \int_{\frac{\theta}{2}}^{\pi} r\cos\left(\frac{\theta}{2} - x_1\right) + r \,\mathrm{d}x_1 \right.\\ &\left. + \int_{\pi}^{2\pi-\frac{\theta}{2}} r\cos\left(\frac{\theta}{2} + x_1\right) + r \,\mathrm{d}x_1 + \int_{2\pi-\frac{\theta}{2}}^{\frac{3\pi}{2}} 2r \,\mathrm{d}x_1 \right) \end{aligned} \quad \text{eqn S3}$$

$$\bar{p}_{\mathrm{NE1}} = \frac{r}{\pi}\left(\theta + 2\sin\left(\frac{\theta}{2}\right)\right) \quad \text{eqn S4}$$

S2.4.2. *Model NE2.* Model NE2 is bounded by $\alpha \leq 3\pi - \theta$, $\alpha \geq 4\pi - 2\theta$ and $\alpha \geq \pi$ (Fig. S2.1). It is the same as NE1 except that the third profile starts at $5\pi/2 - \theta/2 - \alpha/2$ instead of at $\pi$ which is reflected in the different bounds in the second and third integral.

$$\begin{aligned}\bar{p}_{\mathrm{NE2}} =& \frac{1}{\pi}\left( \int_{\frac{\pi}{2}}^{\frac{\theta}{2}} 2r \,\mathrm{d}x_1 + \int_{\frac{\theta}{2}}^{\frac{5\pi}{2}-\frac{\theta}{2}-\frac{\alpha}{2}} r\cos\left(\frac{\theta}{2} - x_1\right) + r \,\mathrm{d}x_1 \right.\\ &\left. + \int_{\frac{5\pi}{2}-\frac{\theta}{2}-\frac{\alpha}{2}}^{2\pi-\frac{\theta}{2}} r\cos\left(\frac{\theta}{2} + x_1\right) + r \,\mathrm{d}x_1 + \int_{2\pi-\frac{\theta}{2}}^{\frac{3\pi}{2}} 2r \,\mathrm{d}x_1 \right) \end{aligned} \quad \text{eqn S5}$$

$$\bar{p}_{\mathrm{NE2}} = \frac{r}{\pi}\left(\theta - \cos\left(\frac{\alpha}{2}\right) + \cos\left(\frac{\alpha}{2} + \theta\right)\right) \quad \text{eqn S6}$$

S2.4.3. *Model NE3.* Model NE3 is bound by $\alpha \leq 4\pi - 2\theta$, $\alpha \geq \pi$ and $\theta \geq \pi$ (Fig. S2.1). It is the same as NE2 except that it contains the extra profile with width $r$ (third integral).

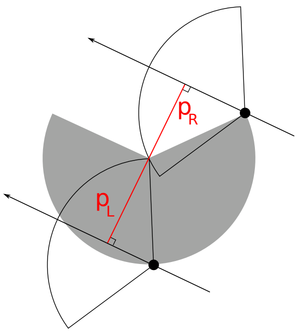


Figure S2.4. The second integral in SE. The right side of the profile ($p_R$) is limited by the size of the sensor region while the left side of the profile ($p_L$) is limited by the size of the signal width. The full profile has width $p = r\sin(\alpha/2) + r\cos(\theta/2 - x_1)$. The sector shaped detection region is shown in grey. Animals are filled black circles and the animal signal is an unfilled sector. The animals direction of movement is indicated with an arrow. The profile $p$ is shown with a red line.

$$\bar{p}_{\mathrm{NE3}} = \frac{1}{\pi}\left( \int_{\frac{\pi}{2}}^{\frac{\theta}{2}} 2r \,\mathrm{d}x_1 + \int_{\frac{\theta}{2}}^{\frac{\theta}{2}+\frac{\pi}{2}} r\cos\left(\frac{\theta}{2} - x_1\right) + r \,\mathrm{d}x_1 + \int_{\frac{\theta}{2}+\frac{\pi}{2}}^{\frac{5\pi}{2}-\frac{\theta}{2}-\frac{\alpha}{2}} r \,\mathrm{d}x_1 + \int_{\frac{5\pi}{2}-\frac{\theta}{2}-\frac{\alpha}{2}}^{2\pi-\frac{\theta}{2}} r\cos\left(\frac{\theta}{2} + x_1\right) + r \,\mathrm{d}x_1 + \int_{2\pi-\frac{\theta}{2}}^{\frac{3\pi}{2}} 2r \,\mathrm{d}x_1 \right) \qquad \text{eqn S7}$$

$$\bar{p}_{\mathrm{NE3}} = \frac{r}{\pi}\left(\theta - \cos\left(\frac{\alpha}{2}\right) + 1\right) \qquad \text{eqn S8}$$

S2.5. **Models SE2–4.** Quadrant SE contains three submodels excluding SE1 (Fig. S2.1). The differences between these three models are similar to the differences between the models in NE. There are four possible profiles.

(1) As $\alpha$ is less than $\pi$ the profile is smaller than $2r$, even when the sensor width is a full diameter. The profile width starts as $2r\sin(\alpha/2)$.
(2) Similar to NE, at a certain point the blind spot of the sensor area limits the profile width on one side. This gives a profile width of $r\sin(\alpha/2) + r\cos(x_1 - \theta/2)$ (Fig. S2.4).
(3) Also similar to NE, there can be a point where the right side of the profile is 0 giving a profile width of $r\sin(\alpha/2)$.
(4) If $\alpha \leq 2\pi - \theta$, then at $x_1 = \theta/2 + \pi/2 + \alpha/2$ the profile width becomes 0. This inequality distinguishes between SE3 and SE4.
(5) The third profile $r\sin(\alpha/2)$ starts at $\theta/2 + \pi/2$ while at $5\pi/2 - \alpha/2 - \theta/2$ the profile returns to size $2r\sin(\alpha/2)$. If $\theta/2 + \pi/2 \geq 5\pi/2 - \alpha/2 - \theta/2$ we go straight into the $2r\sin(\alpha/2)$ profile and miss the $r\sin(\alpha/2)$ profile. SE2 and SE3 are separated by this inequality which simplifies to $\alpha \leq 4\pi - 2\theta$.

S2.5.1. *Model SE2.* SE2 is bounded by $\alpha \geq 4\pi - 2\theta$, $\alpha \leq \pi$ and $\theta \leq 2\pi$ (Fig. S2.1). As $\alpha \geq 4\pi - 2\theta$, there is no $r\sin(\alpha/2)$ profile. As $\alpha \leq 4\pi - 2\theta$, the profile returns to $2r\sin(\alpha/2)$ rather than going to 0. These integrals relate to profiles (1), (2) and (5) in Section S2.5.

$$\bar{p}_{\mathrm{SE2}} = \frac{1}{\pi}\left( \int_{\frac{\pi}{2}}^{\frac{\pi}{2}+\frac{\theta}{2}-\frac{\alpha}{2}} 2r\sin\left(\frac{\alpha}{2}\right) \mathrm{d}x_1 + \int_{\frac{\pi}{2}+\frac{\theta}{2}-\frac{\alpha}{2}}^{\frac{5\pi}{2}-\frac{\theta}{2}-\frac{\alpha}{2}} r\sin\left(\frac{\alpha}{2}\right) + r\cos\left(\frac{\theta}{2} - x_1\right) \mathrm{d}x_1 + \int_{\frac{5\pi}{2}-\frac{\theta}{2}-\frac{\alpha}{2}}^{\frac{3\pi}{2}} 2r\sin\left(\frac{\alpha}{2}\right) \mathrm{d}x_1 \right) \qquad \text{eqn S9}$$

$$\bar{p}_{\mathrm{SE2}} = \frac{r}{\pi}\left(\theta\sin\left(\frac{\alpha}{2}\right) - \cos\left(\frac{\alpha}{2}\right) + \cos\left(\frac{\alpha}{2} + \theta\right)\right) \qquad \text{eqn S10}$$

S2.5.2. *Model SE3.* SE3 is bounded by $4\pi - 2\theta \leq \alpha \leq 4\pi - 2\theta$ and $\alpha \leq \pi$ (Fig. S2.1). Therefore there is a $r\sin(\alpha/2)$ profile but no $0r$ profile. This relates to profiles (1), (2), (3) and (5) above.

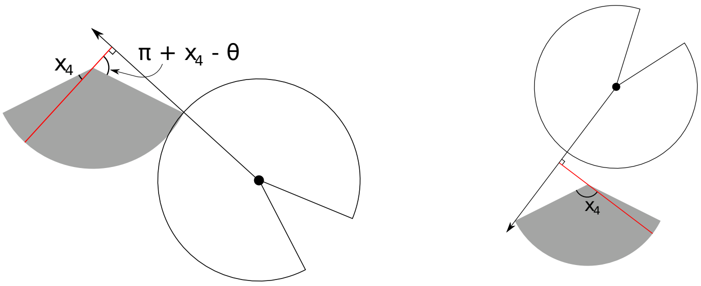


(a) (b)

Figure S2.5. The second and fourth profiles of NW1. The left side of of both profiles is of width $r$ while the right side differs. (a) The right side of the profile is $r\cos(\pi + x_4 - \theta) = -r\cos(\theta - x_4)$ (b) The right side is $r\cos(\pi - x_4) = -r\cos x_4$ respectively. In both images the sector shaped detection region is shown in grey. Animals are filled black circles and the animal signal is an unfilled sector. The animals direction of movement is indicated with an arrow. The profile $p$ is shown with a red line.

$$\bar{p}_{\mathrm{SE3}} = \frac{1}{\pi}\left( \int_{\frac{\pi}{2}}^{\frac{\pi}{2}+\frac{\theta}{2}-\frac{\alpha}{2}} 2r\sin\left(\frac{\alpha}{2}\right)\mathrm{d}x_1 + \int_{\frac{\pi}{2}+\frac{\theta}{2}-\frac{\alpha}{2}}^{\frac{\theta}{2}+\frac{\pi}{2}} r\sin\left(\frac{\alpha}{2}\right) + r\cos\left(\frac{\theta}{2} - x_1\right)\mathrm{d}x_1 + \int_{\frac{\theta}{2}+\frac{\pi}{2}}^{\frac{5\pi}{2}-\frac{\theta}{2}-\frac{\alpha}{2}} r\sin\left(\frac{\alpha}{2}\right)\mathrm{d}x_1 + \int_{\frac{5\pi}{2}-\frac{\theta}{2}-\frac{\alpha}{2}}^{\frac{3\pi}{2}} 2r\sin\left(\frac{\alpha}{2}\right)\mathrm{d}x_1 \right) \qquad \text{eqn S11}$$

$$\bar{p}_{\mathrm{SE3}} = \frac{r}{\pi}\left(\theta\sin\left(\frac{\alpha}{2}\right) - \cos\left(\frac{\alpha}{2}\right) + 1\right) \qquad \text{eqn S12}$$

S2.5.3. *Model SE4.* Finally SE4 is bounded by $\alpha \leq 4\pi - 2\theta$, $\alpha \leq \pi$ and $\theta \leq \pi$ (Fig. S2.1). It is the same as SE3 except that the profile becomes 0 rather than returning to $2r\sin(\alpha/2)$. This relates to profiles (1), (2), (3) and (4) above though profile (4) with width 0 is not shown.

$$\bar{p}_{\mathrm{SE4}} = \frac{1}{\pi}\left( \int_{\frac{\pi}{2}}^{\frac{\pi}{2}+\frac{\theta}{2}-\frac{\alpha}{2}} 2r\sin\left(\frac{\alpha}{2}\right)\mathrm{d}x_1 + \int_{\frac{\pi}{2}+\frac{\theta}{2}-\frac{\alpha}{2}}^{\frac{\theta}{2}+\frac{\pi}{2}} r\sin\left(\frac{\alpha}{2}\right) + r\cos\left(\frac{\theta}{2} - x_1\right)\mathrm{d}x_1 + \int_{\frac{\theta}{2}+\frac{\pi}{2}}^{\frac{\alpha}{2}+\frac{\theta}{2}+\frac{\pi}{2}} r\sin\left(\frac{\alpha}{2}\right)\mathrm{d}x_1 \right) \qquad \text{eqn S13}$$

$$\bar{p}_{\mathrm{SE4}} = \frac{r}{\pi}\left(\theta\sin\left(\frac{\alpha}{2}\right) - \cos\left(\frac{\alpha}{2}\right) + 1\right) \qquad \text{eqn S14}$$

S2.6. **Model NW1.** NW1 is the first model with $\theta < \pi$. Whereas previously the focal angle has always been $x_1$, we now use different focal angles. $x_2$ and $x_3$ correspond to $\gamma_1$ and $\gamma_2$ in (**?**) while $x_4$ is new. They are described in Fig. S2.2b–d.

There are five different profiles in NW1.

(1) $x_2$ has an interval of $[\pi/2, \theta/2]$ which is from the angle of approach being directly towards the sensor until the profile is parallel to the left hand radius of the sensor sector (Fig. S2.2b). During this interval the profile width is $2r\sin(\theta/2)\sin(x_2)$ which is calculated using the equation for the length of a chord . Note that while rotating anti-clockwise (as usual) $x_2$ decreases in size.

(2) From here, we examine focal angle $x_4$ (note that $x_3$ is used in later models, but is not relevant here.) The left side of the profile is a full radius while the right side is limited to $-r\cos(x_4 - \theta)$ (Fig. S2.5a).

(3) At $x_4 = \theta - \pi/2$, the profile is perpendicular to the edge of the sensor area. Here, the right side of the profile is $0r$ giving a profile size of $r$.

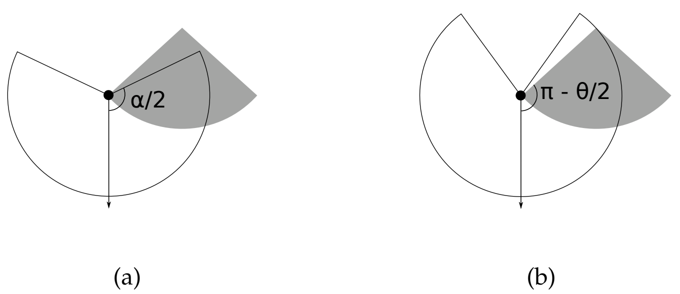


Figure S2.6. Profile sizes when an animal approaches from behind in models NW2–4. If $\alpha$ is relatively large, animals can be detected when approaching from behind. Otherwise animals cannot be detected. The sector shaped detection region is shown in grey. Animals are filled black circles and the animal signal is an unfilled sector. The animals direction of movement is indicated with an arrow. (a) If $\alpha/2$ is less than $\pi - \theta/2$, as is the case here, then the width of the profile when an animal approaches directly from behind is zero. (b) If $\alpha/2 > \pi - \theta/2$ the profile width from behind is $2r\sin(\theta/2)\sin(x_2)$.

(4) When $x_4 = \pi/2$ the angle of approach is from behind the sensor, but we can once again be detected on the right side of the sensor (Fig. S2.5b). Therefore the width of the profile is $r - r\cos(x_4)$.
(5) Finally, we have the $x_2$ profile, but from behind.

$$\bar{p}_{\mathrm{NW1}} = \frac{1}{\pi}\left( \int_{\frac{\theta}{2}}^{\frac{\pi}{2}} 2r\sin\left(\frac{\theta}{2}\right)\sin(x_2)\,\mathrm{d}x_2 + \int_{0}^{\theta-\frac{\pi}{2}} r - r\cos(-x_4+\theta)\,\mathrm{d}x_4 \right.$$
$$\left. + \int_{\theta-\frac{\pi}{2}}^{\frac{\pi}{2}} r\,\mathrm{d}x_4 + \int_{\frac{\pi}{2}}^{\theta} r - r\cos(x_4)\,\mathrm{d}x_4 + \int_{\frac{\theta}{2}}^{\frac{\pi}{2}} 2r\sin\left(\frac{\theta}{2}\right)\sin(x_2)\,\mathrm{d}x_2 \right) \qquad \text{eqn S15}$$

$$\bar{p}_{\mathrm{NW1}} = \frac{r}{\pi}(\theta + 2) \qquad \text{eqn S16}$$

S2.7. **Models NW2–4.** The models NW2–4 have the five potential profiles in NW1 but not all profiles occur in each model, and the angle at which transitions occur are different. Furthermore, there is one extra profile possible.

(1) When approaching the sensor from behind, there is a period where the profile is $r$ wide as in NW1 profile (3).
(2) At some point after profile (1) animals to the right of the sensor can be detected again. If this occurs in the $x_4$ region, the profile width becomes $r - r\cos(x_4)$ as in NW1.
(3) However, as $\alpha$ is now less than $2\pi$, animals to the right of the sensor may be undetectable until we are in the second $x_2$ region. In this case, when we first enter the second $x_2$ region, the profile has a width of $r\cos(x_2 - \theta/2)$. This occurs only if $\alpha \leq 3\pi - 2\theta$. This inequality is found by noting that animals to the right of the sensor can be detected again at $x_4 = 3\pi/2 - \alpha$ but the $x_2$ region starts at $x_4 = \theta$. The new profile in $x_2$ will only occur if $\theta < 3\pi/2 - \alpha/2$ which is rearranged to find the inequality above. This defines the boundary between NW2 and NW3.
(4) As $\alpha \leq 2\pi$ it is possible that when the angle of approach is from directly behind the sensor the animal will not be detected at all. This is the case if $\alpha/2 \leq \pi - \theta/2$ (Fig. S2.6). This inequality (simplified as $\alpha \leq 2\pi - \theta$) defines the boundary between NW3 and NW4.

S2.7.1. *Model NW2.* NW2 is bounded by $\alpha \geq 3\pi - 2\theta$, $\alpha \leq 2\pi$ and $\theta \leq \pi$ (Fig. S2.1).

NW2 has all five profiles as found in NW1. However, the change from the $r$ profile (third integral) to the $r - r\cos(x_4)$ profile (fourth integral) occurs at $x_4 = 3\pi/2 - \alpha/2$ instead of at $x_4 = \theta$.

$$\bar{p}_{\text{NW2}} = \frac{1}{\pi}\left( \int_{\frac{\theta}{2}}^{\frac{\pi}{2}} 2r \sin\left(\frac{\theta}{2}\right) \sin(x_2)\, \mathrm{d}x_2 + \int_{0}^{\theta-\frac{\pi}{2}} r - r\cos(-x_4+\theta)\, \mathrm{d}x_4 \right.$$
$$\left. + \int_{\theta-\frac{\pi}{2}}^{\frac{3\pi}{2}-\frac{\alpha}{2}} r\, \mathrm{d}x_4 + \int_{\frac{3\pi}{2}-\frac{\alpha}{2}}^{\theta} r - r\cos(x_4)\, \mathrm{d}x_4 + \int_{\frac{\theta}{2}}^{\frac{\pi}{2}} 2r\sin\left(\frac{\theta}{2}\right)\sin(x_2)\, \mathrm{d}x_2 \right) \qquad \text{eqn S17}$$

$$\bar{p}_{\text{NW2}} = \frac{r}{\pi}\left(\theta - \cos\left(\frac{\alpha}{2}\right) + 1\right) \qquad \text{eqn S18}$$

S2.7.2. *Model NW3.* NW3 is bounded by $\alpha \leq 3\pi - 2\theta$, $\alpha \geq 2\pi - \theta$ and $\theta \geq \pi/2$ (Fig. S2.1).

NW3 does not have the fourth integral from NW2 as animals are not detectable to the right of the sensor until after the $x_4$ region has ended and the $x_2$ region has begun. Therefore the second $x_4$ integral has an upper limit of $\theta$ and the profile after has a width of $r\cos(x_2-\theta/2)$ and is integrated with respect to $x_2$. The final integral starts at $x_4 = 3\pi/2-\alpha/2-\theta/2$ and has the full width of $2r\sin(x_2)\sin(\theta/2)$.

$$\bar{p}_{\text{NW3}} = \frac{1}{\pi}\left( \int_{\frac{\theta}{2}}^{\frac{\pi}{2}} 2r \sin\left(\frac{\theta}{2}\right) \sin(x_2)\, \mathrm{d}x_2 + \int_{0}^{\theta-\frac{\pi}{2}} r - r\cos(-x_4+\theta)\, \mathrm{d}x_4 \right.$$
$$\left. + \int_{\theta-\frac{\pi}{2}}^{\theta} r\, \mathrm{d}x_4 + \int_{\frac{\theta}{2}}^{\frac{3\pi}{2}-\frac{\theta}{2}-\frac{\alpha}{2}} r\cos\left(\frac{\theta}{2}-x_2\right) \mathrm{d}x_2 + \int_{\frac{3\pi}{2}-\frac{\theta}{2}-\frac{\alpha}{2}}^{\frac{\pi}{2}} 2r\sin\left(\frac{\theta}{2}\right)\sin(x_2)\, \mathrm{d}x_2 \right) \qquad \text{eqn S19}$$

$$\bar{p}_{\text{NW3}} = \frac{r}{\pi}\left(\theta - \cos\left(\frac{\alpha}{2}\right) + 1\right) \qquad \text{eqn S20}$$

S2.7.3. *Model NW4.* Finally, NW4 is bounded by $\alpha \geq \pi$, $\theta \geq \pi/2$ and $\alpha \leq 2\pi - \theta$ (Fig. S2.1). NW4 is the same as NW3 except that the final profile width is zero and this profile is reached at $\alpha/2 + \theta/2 - \pi/2$.

$$\bar{p}_{\text{NW4}} = \frac{1}{\pi}\left( \int_{\frac{\theta}{2}}^{\frac{\pi}{2}} 2r \sin\left(\frac{\theta}{2}\right) \sin(x_2)\, \mathrm{d}x_2 + \int_{0}^{\theta-\frac{\pi}{2}} r - r\cos(-x_4+\theta)\, \mathrm{d}x_4 \right.$$
$$\left. + \int_{\theta-\frac{\pi}{2}}^{\theta} r\, \mathrm{d}x_4 + \int_{\frac{\theta}{2}}^{\frac{\alpha}{2}+\frac{\theta}{2}-\frac{\pi}{2}} r\cos\left(\frac{\theta}{2}-x_2\right) \mathrm{d}x_2 \right) \qquad \text{eqn S21}$$

$$\bar{p}_{\text{NW4}} = \frac{r}{\pi}\left(\theta - \cos\left(\frac{\alpha}{2}\right) + 1\right) \qquad \text{eqn S22}$$

S2.8. **Model REM.** REM is the model from (**?**). It has $\alpha = 2\pi$ and $\theta \leq \pi/2$ (Fig. S2.1). It has three profile widths, two of which are repeated, once as the animal approaches from in front of the sensor and once as the animal approaches from behind the sensor.

(1) Starting with an approach direction of directly towards the sensor, and examining focal angle $x_2$, the profile width is $2r\sin(x_2)\sin(\theta/2)$.
(2) When the profile is perpendicular to the radius on the right hand of the sector sensor region, we instead examine $x_3$ where the profile width is $r\sin(x_3)$.
(3) At $x_3 = \pi/2$ the profile becomes simply $r$ and this continues for $\theta$ radians of $x_4$.
(4) The $x_3$ profile is then repeated with an approach direction from behind the sensor.
(5) Finally the $x_2$ profile is repeated, again with an approach direction from behind the sensor.

$$\bar{p}_{\mathrm{REM}} = \frac{1}{\pi}\left( \int_{\frac{\pi}{2}-\frac{\theta}{2}}^{\frac{\pi}{2}} 2r\sin\left(\frac{\theta}{2}\right)\sin(x_2)\,\mathrm{d}x_2 + \int_{\theta}^{\frac{\pi}{2}} r\sin(x_3)\,\mathrm{d}x_3 \right.$$
$$\left. + \int_{0}^{\theta} r\,\mathrm{d}x_4 + \int_{\theta}^{\frac{\pi}{2}} r\sin(x_3)\,\mathrm{d}x_3 + \int_{\frac{\pi}{2}-\frac{\theta}{2}}^{\frac{\pi}{2}} 2r\sin\left(\frac{\theta}{2}\right)\sin(x_2)\,\mathrm{d}x_2 \right) \quad \text{eqn S23}$$

$$\bar{p}_{\mathrm{REM}} = \frac{r}{\pi}(\theta + 2) \quad \text{eqn S24}$$

S2.9. **Models NW5–7.** In the models NW5–7, the sensor has $\theta \leq \pi/2$ as in the REM. As $\alpha \geq \pi$ a lot of the profiles are similar to the REM. Specifically, the first three profiles are always the same as the first three profiles of the REM. This is because when an animal is moving towards the sensor, the $\alpha \geq \pi$ signal is no different to a $2\pi$ signal. However, when approaching the sensor from behind, things are slightly different. The animal can only be detected by the sensor if the signal width is large enough that it can be detected once it has passed the sensor.

(1) Starting with an approach direction of directly towards the sensor, and examining focal angle $x_2$, the profile width is $2r\sin(x_2)\sin(\theta/2)$.
(2) When the profile is perpendicular to the radius edge of the sector sensor region, we instead examine $x_3$ where the profile width is $r\sin(x_3)$.
(3) At $x_3 = \pi/2$ the profile becomes simply $r$ and this continues for $\theta$ radians of $x_4$.
(4) If $\alpha \leq 2\pi + 2\theta$, the animal becomes undetectable during this profile when $x_3$ has decreased in size to $\pi - \alpha/2$. This inequality marks the boundary between NW7 and NW6.
(5) If instead $\alpha \geq 2\pi + 2\theta$ then the animal does not become undetectable during the $x_3$ focal angle. Instead the profile has width greater than zero for the whole of the $x_3$ angle. The $x_2$ profile starts with width $r\cos(x_2 - \theta/2)$ as only animals approaching to the left of the sensor are detectable.
(6) During this second $x_2$ profile the signal width needed for animals to be detected to the left of the detector is increasing while the angle needed for animals to be detected to the right of the detector is decreasing. Therefore, either the left side becomes undetectable, making both sides undetectable (this occurs if $\alpha \leq 2\pi - \theta$ as in NW6)
(7) or the right becomes detectable (if $\alpha \geq 2\pi - \theta$ as in NW5), making both sides detectable and giving a profile width of $2r\sin(x_2)\sin(\theta/2)$.

S2.9.1. *Model NW5.* NW5 is bounded by $\alpha \geq 2\pi - \theta$, $\alpha \leq 2\pi$ and $\theta \leq \pi/2$ (Fig. S2.1).

It is the same as REM except that it includes the extra profile in $x_2$ (the fifth integral) where only animals approaching to the left of the profile are detected.

$$\bar{p}_{\mathrm{NW5}} = \frac{1}{\pi}\left( \int_{\frac{\pi}{2}-\frac{\theta}{2}}^{\frac{\pi}{2}} 2r\sin\left(\frac{\theta}{2}\right)\sin(x_2)\,\mathrm{d}x_2 + \int_{\theta}^{\frac{\pi}{2}} r\sin(x_3)\,\mathrm{d}x_3 + \int_{0}^{\theta} r\,\mathrm{d}x_4 \right.$$
$$\left. + \int_{\theta}^{\frac{\pi}{2}} r\sin(x_3)\,\mathrm{d}x_3 + \int_{\frac{\pi}{2}-\frac{\theta}{2}}^{\frac{3\pi}{2}-\frac{\theta}{2}-\frac{\alpha}{2}} r\cos\left(\frac{\theta}{2} - x_2\right)\mathrm{d}x_2 + \int_{\frac{3\pi}{2}-\frac{\theta}{2}-\frac{\alpha}{2}}^{\frac{\pi}{2}} 2r\sin\left(\frac{\theta}{2}\right)\sin(x_2)\,\mathrm{d}x_2 \right) \quad \text{eqn S25}$$

$$\bar{p}_{\mathrm{NW5}} = \frac{r}{\pi}\left(\theta - \cos\left(\frac{\alpha}{2}\right) + 1\right) \quad \text{eqn S26}$$

S2.9.2. *Model NW6.* NW6 is bounded by $\alpha \leq 2\pi - \theta$, $\alpha \geq 2\pi + 2\theta$ and $\theta \leq \pi/2$ (Fig. S2.1).

NW6 is the same NW5 except that as $\alpha \leq 2\pi - \theta$, animals that approach from directly behind the detector are not detected. Therefore at $x_2 = \alpha/2 + \theta/2 - \pi/2$ the profile width goes to zero and therefore the last integral in NW5 is not included.

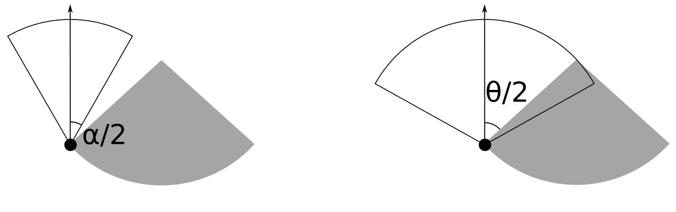


(a) (b)

Figure S2.7. The first profile in SW models is limited by either $\alpha$ or $\beta$ depending on whether $\alpha < \beta$. The sector shaped detection region is shown in grey. Animals are filled black circles and the animal signal is an unfilled sector. The animals direction of movement is indicated with an arrow. (a) As $\alpha/2 < \theta/2$ the profile width is limited by the signal width rather than the sensor region. The profile width is $2r\sin(\alpha/2)$ (b) As $\alpha/2 > \theta/2$ the profile width is limited by the sensor region, not the signal width. The profile width is $2r\sin(\theta/2)\sin(x_2)$.

$$\begin{aligned}\bar{p}_{\mathrm{NW6}} =& \frac{1}{\pi}\Bigg( \int_{\frac{\pi}{2}-\frac{\theta}{2}}^{\frac{\pi}{2}} 2r\sin\left(\frac{\theta}{2}\right)\sin(x_2)\,\mathrm{d}x_2 + \int_{\theta}^{\frac{\pi}{2}} r\sin(x_3)\,\mathrm{d}x_3 \\ &+ \int_0^{\theta} r\,\mathrm{d}x_4 + \int_{\theta}^{\frac{\pi}{2}} r\sin(x_3)\,\mathrm{d}x_3 + \int_{\frac{\pi}{2}-\frac{\theta}{2}}^{\frac{\alpha}{2}+\frac{\theta}{2}-\frac{\pi}{2}} r\cos\left(\frac{\theta}{2}-x_2\right)\mathrm{d}x_2\Bigg) \qquad \text{eqn S27}\end{aligned}$$

$$\bar{p}_{\mathrm{NW6}} = \frac{r}{\pi}\left(\theta - \cos\left(\frac{\alpha}{2}\right) + 1\right) \qquad \text{eqn S28}$$

S2.9.3. *Model NW7.* NW7 is bounded by $\alpha \geq 2\pi + 2\theta$, $\alpha \geq \pi$ and $\theta \geq 0$ (Fig. S2.1).

It is similar to NW6 but does not include the last integral as during the $x_3$ profile, at $x_3 = \pi - \alpha/2$ the signal width is too small for any animals to be detected, so the profile width goes to zero.

$$\begin{aligned}\bar{p}_{\mathrm{NW7}} =& \frac{1}{\pi}\Bigg( \int_{\frac{\pi}{2}-\frac{\theta}{2}}^{\frac{\pi}{2}} 2r\sin\left(\frac{\theta}{2}\right)\sin(x_2)\,\mathrm{d}x_2 + \int_{\theta}^{\frac{\pi}{2}} r\sin(x_3)\,\mathrm{d}x_3 \\ &+ \int_0^{\theta} r\,\mathrm{d}x_4 + \int_{\pi-\frac{\alpha}{2}}^{\frac{\pi}{2}} r\sin(x_3)\,\mathrm{d}x_3\Bigg) \qquad \text{eqn S29}\end{aligned}$$

$$\bar{p}_{\mathrm{NW7}} = \frac{r}{\pi}\left(\theta - \cos\left(\frac{\alpha}{2}\right) + 1\right) \qquad \text{eqn S30}$$

S2.10. **Model SW1–3.** The models in SW1–3 are described with the two focal angles used in models NW2–4, $x_2$ and $x_4$. As $\alpha \leq \pi$ an animal can never be detected if it is approaching the detector from behind. This makes these models simpler in that they go through the $x_2$ and $x_4$ profiles only once each.

There are five potential profile sizes.

(1) At the beginning of $x_2$, with an approach direction directly towards the sensor, the parameter that limits the width of the profile can either be the sensor width, in which case the profile width is $2r\sin(\theta/2)\sin(x_2)$.
(2) Or the signal width can be the limiting parameter, in which case the profile width is instead $2r\sin(\alpha/2)$ (Fig. S2.7)
(3) The next potential profile in $x_2$ has a width of $r\sin(\alpha/2) - r\cos(x_2 + \theta/2)$ as the right side of the profile is limited by the width of the sensor region while the left side is limited by the signal width. However, the angle at which the profile starts depends on whether the first profile was 1) or 2) above. If the first profile is profile 1) then the profile is limited on both sides by the sensor region and then the left side of the profile becomes limited by the signal width. This happens at $x_2 = \pi/2 - \alpha/2 + \theta/2$. If however the first profile was 2) then the first profile is

limited by the signal width. We move into the new profile when the right side of the profile becomes limited by the sensor region. This occurs at $x_2 = \pi/2 + \alpha/2 - \theta/2$.

(4) In the $x_4$ region the left side of the profile is always $r\sin(\alpha/2)$ while the right side is either 0, giving a profile of $r\sin(\alpha/2)$.

(5) Or limited by the sensor giving a profile of size $r\sin(\alpha/2) - r\cos(x_4 - \theta)$.

S2.10.1. *Model SW1.* SW1 is bounded by $\alpha \geq \theta$, $\alpha \leq \pi$ and $\theta \leq \pi$ (Fig. S2.1).

As $\alpha$ is large the first profile is limited by the size of the sensor region giving it a width of $2r\sin(\theta/2)\sin(x_2)$. It is the only one of the three SW models to start in this way. Later on, still with $x_2$ as the focal angle the left side of the profile does become limited by the signal width. So at $x_2 = \pi/2 - \alpha/2 + \theta/2$ the profile width becomes $r\sin(\alpha/2) - r\cos(x_2 + \theta/2)$.

As we enter the $x_4$ region, the profile remains limited by the signal on the left and by the sensor on the right, giving a profile width of $r\sin(\alpha/2) - r\cos(x_4 - \theta)$. Finally, at $x_4 = \theta - \pi/2$ the right side of the profile becomes zero and the profile is width is $r\sin(\alpha/2)$.

$$\begin{aligned}\bar{p}_{\text{SW1}} =& \frac{1}{\pi}\left( \int_{\frac{\pi}{2}+\frac{\theta}{2}-\frac{\alpha}{2}}^{\frac{\pi}{2}} 2r\sin\left(\frac{\theta}{2}\right)\sin(x_2)\,\mathrm{d}x_2 + \int_{\frac{\theta}{2}}^{\frac{\pi}{2}+\frac{\theta}{2}-\frac{\alpha}{2}} r\sin\left(\frac{\alpha}{2}\right) - r\cos\left(\frac{\theta}{2}+x_2\right)\mathrm{d}x_2 \right.\\ &\left. + \int_0^{\theta-\frac{\pi}{2}} r\sin\left(\frac{\alpha}{2}\right) - r\cos(\theta - x_4)\,\mathrm{d}x_4 + \int_{\theta-\frac{\pi}{2}}^{\frac{\alpha}{2}+\theta-\frac{\pi}{2}} r\sin\left(\frac{\alpha}{2}\right)\mathrm{d}x_4 \right) \qquad \text{eqn S31}\end{aligned}$$

$$\bar{p}_{\text{SW1}} = \frac{r}{\pi}\left(\theta\sin\left(\frac{\alpha}{2}\right) - \cos\left(\frac{\alpha}{2}\right) + 1\right) \qquad \text{eqn S32}$$

S2.10.2. *Model SW2.* SW2 is bounded by $\theta \geq \pi/2$, $\alpha \leq \theta$ and $\alpha \geq 2\theta - \pi$ (Fig. S2.1).

SW2 is largely similar to SW1. However, as $\alpha \leq \theta$ the first profile is limited by $\alpha$ and not by the detection region. Therefore the first profile has width $2r\sin(\alpha/2)$. This also means the transition to the second profile occurs at $x_2 = \pi/2 + \alpha/2 - \theta/2$ instead of $x_2 = \pi/2 - \alpha/2 + \theta/2$.

$$\begin{aligned}\bar{p}_{\text{SW2}} =& \frac{1}{\pi}\left( \int_{\frac{\alpha}{2}-\frac{\theta}{2}+\frac{\pi}{2}}^{\frac{\pi}{2}} 2r\sin\left(\frac{\alpha}{2}\right)\mathrm{d}x_2 + \int_{\frac{\theta}{2}}^{\frac{\alpha}{2}-\frac{\theta}{2}+\frac{\pi}{2}} r\sin\left(\frac{\alpha}{2}\right) - r\cos\left(\frac{\theta}{2}+x_2\right)\mathrm{d}x_2 \right.\\ &\left. + \int_0^{\theta-\frac{\pi}{2}} r\sin\left(\frac{\alpha}{2}\right) - r\cos(\theta - x_4)\,\mathrm{d}x_4 + \int_{\theta-\frac{\pi}{2}}^{\frac{\alpha}{2}+\theta-\frac{\pi}{2}} r\sin\left(\frac{\alpha}{2}\right)\mathrm{d}x_4 \right) \qquad \text{eqn S33}\end{aligned}$$

$$\bar{p}_{\text{SW2}} = \frac{r}{\pi}\left(\theta\sin\left(\frac{\alpha}{2}\right) - \cos\left(\frac{\alpha}{2}\right) + 1\right) \qquad \text{eqn S34}$$

S2.10.3. *Model SW3.* SW3 is bounded by $\alpha \leq 2\theta - \pi$ and $\theta \leq \pi$ (Fig. S2.1).

SW3 is similar to SW2 except that the profile does not become limited by sensor at all during the the $x_4$ regions. Therefore, at $x_4 = 0$ the profile is still of width $2r\sin(\alpha/2)$. Only at $x_4 = \theta - \pi/2 - \alpha/2$ does the profile become limited on the right by the sensor region.

$$\begin{aligned}\bar{p}_{\text{SW3}} =& \frac{1}{\pi}\left( \int_{\frac{\theta}{2}}^{\frac{\pi}{2}} 2r\sin\left(\frac{\alpha}{2}\right)\mathrm{d}x_2 + \int_0^{-\frac{\pi}{2}+\theta-\frac{\alpha}{2}} 2r\sin\left(\frac{\alpha}{2}\right)\mathrm{d}x_4 \right.\\ &\left. + \int_{-\frac{\pi}{2}+\theta-\frac{\alpha}{2}}^{\theta-\frac{\pi}{2}} r\sin\left(\frac{\alpha}{2}\right) - r\cos(\theta - x_4)\,\mathrm{d}x_4 + \int_{\theta-\frac{\pi}{2}}^{\frac{\alpha}{2}+\theta-\frac{\pi}{2}} r\sin\left(\frac{\alpha}{2}\right)\mathrm{d}x_4 \right) \qquad \text{eqn S35}\end{aligned}$$

$$\bar{p}_{\text{SW3}} = \frac{r}{\pi}\left(\theta\sin\left(\frac{\alpha}{2}\right) - \cos\left(\frac{\alpha}{2}\right) + 1\right) \qquad \text{eqn S36}$$

S2.11. **Model SW4–9.** As $\alpha < \pi$, animals approaching the sensor from behind can never be detected, so unlike REM, the second $x_2$ and $x_3$ profiles are always zero. The six models are split by three inequalities that relate to the models as follows.

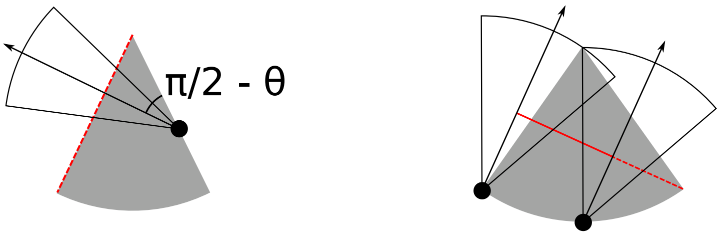


(a) (b)

Figure S2.8. Description of two profiles in SW models. The sector shaped detection region is shown in grey. Animals are filled black circles and the animal signal is an unfilled sector. The animals direction of movement is indicated with an arrow. The profile $p$ is shown with a red line. Dashed red lines indicate areas where animals cannot be detected. (a) At $x_4 = 0$, if $\alpha/2 < \pi/2 - \theta$ then $\alpha/2$ is too small for an animal to be detected at all during the $x_4$ profile (shown with dashed red.) This inequality simplifies to $\alpha < \pi - 2\theta$. (b) The right of the profile is limited by the signal width, not the sensor. On the left, the profile is limited by the sensor and not the signal. Overall the profile width is $r\sin(\alpha/2) - r\cos(x_2 + \theta/2)$.

(1) Models with $\alpha \leq \pi - 2\theta$ have no $x_4$ profile. This is because at $x_4 = 0$, the signal width is already too small to be detected as can be seen in Fig. S2.8a where $\alpha/2 < \pi/2 - \theta$ which simplifies to give the previous inequality.
(2) Models with $\alpha \leq \theta$ are limited by $\alpha$ in the first, $x_2$ region (Fig. S2.7), rather than being limited by $\theta$. Therefore this first profile is of width $2r\sin(\alpha/2)$ rather than $2r\sin(\theta/2)\sin(x_2)$.
(3) Finally, models with $\alpha \leq 2\theta$ have a second profile in $x_2$ where to one side of the sensor $\alpha$ is the limiting factor of profile width, while on the other side $\theta$ is (Fig. S2.8b). This gives a width of $r\sin(\alpha/2) - r\cos(x_2 + \theta/2)$. This profile does not occur in models with $\alpha \geq 2\theta$.

S2.11.1. *Model SW4.* SW4 is bounded by $\alpha \leq \theta$, $\alpha \geq \pi - 2\theta$ and $\theta \leq \pi/2$ (Fig. S2.1). Therefore it does contain a $x_4$ profile, starts with an $\alpha$ limited profile and does contain the $r\sin(\alpha/2) - r\cos(x_2 + \theta/2)$ profile in $x_2$.

$$\bar{p}_{\text{SW4}} = \frac{1}{\pi}\left( \int_{\frac{\alpha}{2}-\frac{\theta}{2}+\frac{\pi}{2}}^{\frac{\pi}{2}} 2r\sin\left(\frac{\alpha}{2}\right)\mathrm{d}x_2 + \int_{\frac{\pi}{2}-\frac{\theta}{2}}^{\frac{\alpha}{2}-\frac{\theta}{2}+\frac{\pi}{2}} r\sin\left(\frac{\alpha}{2}\right) - r\cos\left(\frac{\theta}{2}+x_2\right)\mathrm{d}x_2 + \int_{\theta}^{\frac{\pi}{2}} r\sin\left(\frac{\alpha}{2}\right)\mathrm{d}x_3 + \int_{0}^{\frac{\alpha}{2}+\theta-\frac{\pi}{2}} r\sin\left(\frac{\alpha}{2}\right)\mathrm{d}x_4 \right) \quad \text{eqn S37}$$

$$\bar{p}_{\text{SW4}} = \frac{r}{\pi}\left(\theta\sin\left(\frac{\alpha}{2}\right) - \cos\left(\frac{\alpha}{2}\right) + 1\right) \quad \text{eqn S38}$$

S2.11.2. *Model SW5.* SW5 is the only model with a tetrahedral bounding region. It is bounded by $\alpha \geq \theta$, $\alpha \geq \pi - 2\theta$, $\alpha \leq 2\theta$ and $\theta \leq \pi/2$ (Fig. S2.1). Therefore it does contain a $x_4$ profile, but starts with a $\theta$ limited profile. It does contain the $r\sin(\alpha/2) - r\cos(x_2 + \theta/2)$ profile in $x_2$.

$$\bar{p}_{\text{SW5}} = \frac{1}{\pi}\left( \int_{\frac{\pi}{2}+\frac{\theta}{2}-\frac{\alpha}{2}}^{\frac{\pi}{2}} 2r\sin\left(\frac{\theta}{2}\right)\sin(x_2)\,\mathrm{d}x_2 + \int_{\frac{\pi}{2}-\frac{\theta}{2}}^{\frac{\pi}{2}+\frac{\theta}{2}-\frac{\alpha}{2}} r\sin\left(\frac{\alpha}{2}\right) - r\cos\left(\frac{\theta}{2}+x_2\right)\mathrm{d}x_2 + \int_{\theta}^{\frac{\pi}{2}} r\sin\left(\frac{\alpha}{2}\right)\mathrm{d}x_3 + \int_{0}^{\frac{\alpha}{2}+\theta-\frac{\pi}{2}} r\sin\left(\frac{\alpha}{2}\right)\mathrm{d}x_4 \right) \quad \text{eqn S39}$$

$$\bar{p}_{\text{SW5}} = \frac{r}{\pi}\left(\theta\sin\left(\frac{\alpha}{2}\right) - \cos\left(\frac{\alpha}{2}\right) + 1\right) \quad \text{eqn S40}$$

S2.11.3. *Model SW6.* SW6 is bounded by $\alpha \geq \pi - 2\theta$, $\alpha \geq 2\theta$ and $\alpha \leq \pi$ (Fig. S2.1). It starts with a $\theta$ limited profile and has a $x_4$ profile. However, it does not contain the $r\sin(\alpha/2) - r\cos(x_2 + \theta/2)$ profile.

$$\bar{p}_{\text{SW6}} = \frac{1}{\pi}\left( \int_{\frac{\pi}{2}-\frac{\theta}{2}}^{\frac{\pi}{2}} 2r\sin\left(\frac{\theta}{2}\right)\sin(x_2)\,\mathrm{d}x_2 + \int_{\theta}^{\frac{\alpha}{2}} r\sin(x_3)\,\mathrm{d}x_3 + \int_{\frac{\alpha}{2}}^{\frac{\pi}{2}} r\sin\left(\frac{\alpha}{2}\right)\mathrm{d}x_3 + \int_{0}^{\frac{\alpha}{2}+\theta-\frac{\pi}{2}} r\sin\left(\frac{\alpha}{2}\right)\mathrm{d}x_4 \right) \quad \text{eqn S41}$$

$$\bar{p}_{\text{SW6}} = \frac{r}{\pi}\left(\theta\sin\left(\frac{\alpha}{2}\right) - \cos\left(\frac{\alpha}{2}\right) + 1\right) \quad \text{eqn S42}$$

S2.11.4. *Model SW7.* SW7 is bounded by $\alpha \leq \pi - 2\theta$, $\alpha \leq \theta$ and $\alpha < 0$ (Fig. S2.1). Therefore it does not contain a $x_4$ profile. It starts with an $\alpha$ limited profile and contains the $r\sin(\alpha/2) - r\cos(x_2 + \theta/2)$ profile in $x_2$.

$$\bar{p}_{\text{SW7}} = \frac{1}{\pi}\left( \int_{\frac{\alpha}{2}-\frac{\theta}{2}+\frac{\pi}{2}}^{\frac{\pi}{2}} 2r\sin\left(\frac{\alpha}{2}\right)\mathrm{d}x_2 + \int_{\frac{\pi}{2}-\frac{\theta}{2}}^{\frac{\alpha}{2}-\frac{\theta}{2}+\frac{\pi}{2}} r\sin\left(\frac{\alpha}{2}\right) - r\cos\left(\frac{\theta}{2} + x_2\right)\mathrm{d}x_2 + \int_{\theta}^{\frac{\alpha}{2}+\theta} r\sin\left(\frac{\alpha}{2}\right)\mathrm{d}x_3 \right) \quad \text{eqn S43}$$

$$\bar{p}_{\text{SW7}} = \frac{r}{\pi}\left(\theta\sin\left(\frac{\alpha}{2}\right) - \cos\left(\frac{\alpha}{2}\right) + 1\right) \quad \text{eqn S44}$$

S2.11.5. *Model SW8.* SW8 is bounded by $\alpha \leq \pi - 2\theta$, $\alpha \geq \theta$ and $\alpha \leq 2\theta$ (Fig. S2.1). It starts with a $\theta$ limited profile. It does contain the $r\sin(\alpha/2) - r\cos(x_2 + \theta/2)$ profile in $x_2$ but does not have a $x_4$ profile.

$$\bar{p}_{\text{SW8}} = \frac{1}{\pi}\left( \int_{\frac{\pi}{2}+\frac{\theta}{2}-\frac{\alpha}{2}}^{\frac{\pi}{2}} 2r\sin\left(\frac{\theta}{2}\right)\sin(x_2)\,\mathrm{d}x_2 + \int_{\frac{\pi}{2}-\frac{\theta}{2}}^{\frac{\pi}{2}+\frac{\theta}{2}-\frac{\alpha}{2}} r\sin\left(\frac{\alpha}{2}\right) - r\cos\left(\frac{\theta}{2} + x_2\right)\mathrm{d}x_2 + \int_{\theta}^{\frac{\alpha}{2}+\theta} r\sin\left(\frac{\alpha}{2}\right)\mathrm{d}x_3 \right) \quad \text{eqn S45}$$

$$\bar{p}_{\text{SW8}} = \frac{r}{\pi}\left(\theta\sin\left(\frac{\alpha}{2}\right) - \cos\left(\frac{\alpha}{2}\right) + 1\right) \quad \text{eqn S46}$$

S2.11.6. *Model SW9.* Finally, SW9, the last model, is bounded by y $\alpha \leq \pi - 2\theta$, $\alpha \geq 2\theta$ and $\theta \geq 0$ (Fig. S2.1). Therefore it starts with a $\theta$ limited profile. However it does not contain the extra $x_2$ profile nor a $x_4$ profile.

$$\bar{p}_{\text{SW9}} = \frac{1}{\pi}\left( \int_{\frac{\pi}{2}-\frac{\theta}{2}}^{\frac{\pi}{2}} 2r\sin\left(\frac{\theta}{2}\right)\sin(x_2)\,\mathrm{d}x_2 + \int_{\theta}^{\frac{\alpha}{2}} r\sin(x_3)\,\mathrm{d}x_3 + \int_{\frac{\alpha}{2}}^{\frac{\alpha}{2}+\theta} r\sin\left(\frac{\alpha}{2}\right)\mathrm{d}x_3 \right) \quad \text{eqn S47}$$

$$\bar{p}_{\text{SW9}} = \frac{r}{\pi}\left(\theta\sin\left(\frac{\alpha}{2}\right) - \cos\left(\frac{\alpha}{2}\right) + 1\right) \quad \text{eqn S48}$$

## S3. Supplementary Script: Symbolic algebra python script

This script uses the SymPy package (?), a computer algebra system to calculate the equations for $p$ in the various models and to perform unit checks on the results.

The script can also be found in the plain text file `lucas_et_al_S3.py`. It will also be hosted and given a DOI on Github at the time of publication (`https://github.com/timcdlucas/lucasMoorcroftManuscript/tree/postPeerReview`).

```
"""
S3
Supplementary Python script from
A generalised random encounter model for estimating animal density with remote sensor data
Tim C.D. Lucas, Elizabeth A. Moorcroft, Robin Freeman, Marcus J. Rowcliffe, Kate E. Jones
"""

"""
Systematic analysis of REM models
Tim Lucas
01/10/13
"""



"""

This script contains:
1. The integration of all models (lines 24 - 603)
2. Some tests that the models are correct (606 - 738)
3. A python function to calculate \bar{p} given any parameters (743 - 763)
4. Creation of a plot of \bar{p} over all parameter space (767 - 794)
5. Code to create supplementaryS4.R, an R implementation of the model (797 - 873)

"""

from sympy import *
import numpy as np
import matplotlib.pyplot as pl
from datetime import datetime
import os as os

# Set working directory
os.chdir(’supplementary-material’)

# Use LaTeX printing
from sympy import init_printing ;
init_printing()
# Make LaTeX output white. Because I use a dark theme
init_printing(forecolor="White")


# Load symbols used for symbolic maths
t, a, r, x2, x3, x4, x1 = symbols(’theta alpha r x_2 x_3 x_4 x_1’, positive=True)
r1 = {r:1} # useful for lots of checks



# Define functions
# Calculate the final profile averaged over pi.
def calcModel(model):
        x = pi**-1 * sum( [integrate(m[0], m[1:]) for m in model] ).simplify().trigsimp()
        return x

# Do the replacements fit within the area defined by the conditions?
def confirmReplacements(conds, reps):
        if not all([c.subs(reps) for c in eval(conds)]):
                print(’reps’ + conds[4:] + ’ incorrect’)

# is average profile in range 0r-2r?
def profileRange(prof, reps):
        if not 0 <= eval(prof).subs(dict(reps, **r1)) <= 2:
                print(’Total ’ + prof + ’ not in 0, 2r’)

# Are the individuals integrals >0r
def intsPositive(model, reps):
        m = eval(model)
        for i in range(len(m)):
                if not integrate(m[i][0], m[i][1:]).subs(dict(reps, **r1)) > 0:
                    print(’Integral ’ + str(i+1) + ’ in ’ + model + ’ is negative’)

# Are the individual averaged integrals between 0 and  2r
def intsRange(model, reps):
        m = eval(model)
```

```
        for i in range(len(m)):
                if not 0 <= (integrate(m[i][0], m[i][1:])/(m[i][3]-m[i][2])).subs(dict(reps, **r1)) <= 2:
                        print('Integral ' + str(i+1) + ' in ' + model + ' has averaged integral outside 0<p<2r')

# Are the bounds the correct way around
def checkBounds(model, reps):
        m = eval(model)
        for i in range(len(m)):
                if not (m[i][3]-m[i][2]).subs(reps) > 0:
                        print('Bounds ' + str(i+1) + ' in ' + model + ' has lower bounds bigger than upper bounds')

# create latex strings with the 1) the integral equation that defines it and 2)  the final calculated model.
# There's some if statements to split longer equations on two lines and get +s in the right place.
def parseLaTeX(prof):
        m = eval( 'm' + prof[1:] )

        f = open('/latexFiles/'+prof+'.tex', 'w')
        f.write('\\begin{align}\n    \\bar{p}_{\\text{\\tiny{' + prof[1:] + '}}} =&\\frac{1}{\pi} \left(\;\;')
        for i in range(len(m)):
    # Roughly try and prevent expressions beginning with minus signs.
    if latex(m[i][2])[0]=='-':
      o1 = 'rev-lex'
    else:
      o1 = 'lex'

    if latex(m[i][3])[0]=='-':
      o2 = 'rev-lex'
    else:
      o2 = 'lex'

    if latex(m[i][0])[0]=='-':
      o3 = 'rev-lex'
    else:
      o3 = 'lex'

    if latex(m[i][1])[0]=='-':
      o4 = 'rev-lex'
    else:
      o4 = 'lex'

                f.write('\int\limits_{'+latex(m[i][2], order=o1)+'}^{'+latex(m[i][3], order=o2)+'}'+latex(m[i][0], order=o3)+'\;\mathrm{d}' +latex(m[i][1], order=o4))
                if len(m)>3 and i==(len(m)/2)-1:
                        f.write( '\\right.\\notag\\\\\n &\left.' )
                if i<len(m)-1:
                        f.write('+')
        f.write('\\right)\label{' + prof + 'Def}\\\\\n    ')
        f.write('\\bar{p}_{\\text{\\tiny{' + prof[1:] + '}}}  =& ' + latex(eval(prof)) + '\label{' + prof + 'Sln}\n\\end{align}')
        f.close()


# Apply all checks.
def allChecks(prof):
        model = 'm' + prof[1:]
        reps = eval('rep' + prof[1:])
        conds = 'cond' + prof[1:]
        confirmReplacements(conds, reps)
        profileRange(prof, reps)
        intsPositive(model, reps)
        intsRange(model, reps)
        checkBounds(model, reps)

#########################################################
### Define and solve all models                       ###
#########################################################

# NE1 animal: a = 2*pi.  sensor: t > pi, a > 3pi - t  #

mNE1 = [ [2*r,                 x1, pi/2, t/2        ],
         [r + r*cos(x1 - t/2), x1, t/2,  pi         ],
         [r + r*cos(x1 + t/2), x1, pi,   2*pi-t/2   ],
         [2*r,                 x1, 2*pi-t/2, 3*pi/2 ] ]

# Replacement values in range
repNE1 = {t:3*pi/2, a:2*pi}

# Define conditions for model
condNE1 = [pi <= t, a >= 3*pi - t]

# Calculate model, run checks, write output.
pNE1 = calcModel(mNE1)
```

```
allChecks('pNE1')
parseLaTeX('pNE1')


# NE2 animal: a > pi.  sensor: t > pi Condition: a < 3pi - t, a > 4pi - 2t  #

mNE2 = [ [2*r,                  x1, pi/2, t/2          ],
         [r + r*cos(x1 - t/2), x1, t/2,  5*pi/2 - t/2 - a/2 ],
         [r + r*cos(x1 + t/2), x1, 5*pi/2 - t/2 - a/2,   2*pi-t/2 ],
         [2*r,                  x1, 2*pi-t/2, 3*pi/2 ] ]

# Replacement values in range
repNE2 = {t:5*pi/3, a:4*pi/3-0.1}

# Define conditions for model
condNE2 = [pi <= t, a >= pi, a <= 3*pi - t, a >= 4*pi - 2*t]

# Calculate model, run checks, write output.
pNE2 = calcModel(mNE2)
allChecks('pNE2')
parseLaTeX('pNE2')


# NE3 animal: a > pi.  sensor: t > pi Condition: a < 4pi - 2t  #

mNE3 = [ [2*r,                  x1, pi/2, t/2          ],
         [r + r*cos(x1 - t/2), x1, t/2,  t/2 + pi/2          ],
         [r                  , x1, t/2 + pi/2,   5*pi/2 - t/2 - a/2 ],
         [r + r*cos(x1 + t/2), x1, 5*pi/2 - t/2 - a/2,   2*pi-t/2 ],
         [2*r,                  x1, 2*pi-t/2, 3*pi/2 ] ]

# Replacement values in range
repNE3 = {t:5*pi/4-0.1, a:3*pi/2}

# Define conditions for model
condNE3 = [pi <= t, a >= pi, a <= 4*pi - 2*t]

# Calculate model, run checks, write output.
pNE3 = calcModel(mNE3)
allChecks('pNE3')
parseLaTeX('pNE3')


# NW1 animal: a = 2*pi.   sensor:  pi/2 <= t <= pi      #

mNW1 = [ [2*r*sin(t/2)*sin(x2), x2, t/2,      pi/2     ],
        [r - r*cos(x4 - t),      x4, 0,        t - pi/2 ],
        [r,                      x4, t - pi/2, pi/2     ],
        [r - r*cos(x4),          x4, pi/2,     t        ],
        [2*r*sin(t/2)*sin(x2),  x2, t/2,      pi/2     ] ]

# Replacement values in range
repNW1 = {t:3*pi/4}

# Define conditions for model
condNW1 = [pi/2 <= t, t <= pi]

# Calculate model, run checks, write output.
pNW1 = calcModel(mNW1)
allChecks('pNW1')
parseLaTeX('pNW1')




# NW2 animal: a > pi.  Sensor: pi/2 <= t <= pi. Condition: a > 2pi - t          #

mNW2 = [ [2*r*sin(t/2)*sin(x2), x2, t/2,          pi/2        ],
         [r - r*cos(x4 - t),    x4, 0,            t - pi/2    ],
         [r,                    x4, t - pi/2,     3*pi/2 - a/2],
         [r - r*cos(x4),        x4, 3*pi/2 - a/2, t           ],
         [2*r*sin(t/2)*sin(x2), x2, t/2,          pi/2        ] ]


repNW2 = {t:3*pi/4, a:15*pi/8} # Replacement values in range

# Define conditions for model
condNW2 = [a > pi, pi/2 <= t, t <= pi, a >= 3*pi - 2*t]

# Calculate model, run checks, write output.
pNW2 = calcModel(mNW2)
allChecks('pNW2')
parseLaTeX('pNW2')



# NW3 animal: a > pi.  Sensor: pi/2 <= t <= pi. Cond: 2pi - t < a < 3pi - 2t    #
```

```
mNW3 = [ [2*r*sin(t/2)*sin(x2), x2, t/2,                pi/2              ],
         [r - r*cos(x4 - t),    x4, 0,                  t - pi/2          ],
         [r,                    x4, t - pi/2,           t                 ],
         [r*cos(x2 - t/2),      x2, t/2,                3*pi/2 - a/2 - t/2],
         [2*r*sin(t/2)*sin(x2), x2, 3*pi/2 - a/2 - t/2, pi/2              ] ]


repNW3 = {t:5*pi/8, a:6*pi/4} # Replacement values in range

# Define conditions for model
condNW3 = [a > pi, pi/2 <= t, t <= pi, 2*pi - t <= a, a <= 3*pi - 2*t]

# Calculate model, run checks, write output.
pNW3 = calcModel(mNW3)
allChecks('pNW3')
parseLaTeX('pNW3')



# NW4 animal:  a > pi.  Sensor: pi/2 <= t <= pi. Condition: a <= 2pi - t      #

mNW4 = [ [2*r*sin(t/2)*sin(x2), x2, t/2, pi/2],
         [r - r*cos(x4 - t),    x4, 0, t - pi/2],
         [r,                    x4, t - pi/2, t],
         [r*cos(x2 - t/2),      x2, t/2, a/2 + t/2 - pi/2] ]

repNW4 = {t:3*pi/4, a:9*pi/8} # Replacement values in range

# Define conditions for model
condNW4 = [a > pi,  pi/2 <= t, t <= pi, a <= 2*pi - t]

# Calculate model, run checks, write output.
pNW4 = calcModel(mNW4)
allChecks('pNW4')
parseLaTeX('pNW4')


# REM animal: a=2pi.  Sensor: t <= pi/2.                                      #

mREM = [ [2*r*sin(t/2)*sin(x2), x2, pi/2 - t/2, pi/2],
         [r*sin(x3),            x3, t,          pi/2],
         [r,                    x4, 0*t,          t],
         [r*sin(x3),            x3, t,          pi/2],
         [2*r*sin(t/2)*sin(x2), x2, pi/2 - t/2, pi/2] ]


repREM = {t:3*pi/8, a:2*pi} # Replacement values in range

# Define conditions for model
condREM = [ t <= pi/2  ]

# Calculate model, run checks, write output.
pREM = calcModel(mREM)
allChecks('pREM')
parseLaTeX('pREM')



# NW5 animal: a>pi.  Sensor: t <= pi/2. Condition: 2*pi - t < a              #


mNW5 = [ [2*r*sin(t/2)*sin(x2), x2, pi/2 - t/2, pi/2],
         [r*sin(x3),            x3, t,          pi/2],
         [r,                    x4, 0,          t],
         [r*sin(x3),            x3, t,          pi/2],
         [r*cos(x2 - t/2),      x2, pi/2 - t/2, 3*pi/2 - t/2 - a/2],
         [2*r*sin(t/2)*sin(x2), x2, 3*pi/2 - t/2 - a/2, pi/2] ]


repNW5 = {t:3*pi/8, a:29*pi/16} # Replacement values in range

# Define conditions for model
condNW5 = [a >= pi, t <= pi/2, 2*pi - t <= a  ]

# Calculate model, run checks, write output.
pNW5 = calcModel(mNW5)
allChecks('pNW5')
parseLaTeX('pNW5')


# NW6 animal: a>pi.  Sensor: t <= pi/2. Condition:  2*pi - 2*t <= a <= 2*pi - t  #


mNW6 = [ [2*r*sin(t/2)*sin(x2), x2, pi/2 - t/2, pi/2],
         [r*sin(x3),            x3, t,          pi/2],
         [r,                    x4, 0,          t],
```

```
         [r*sin(x3),            x3, t,          pi/2],
         [r*cos(x2 - t/2),      x2, pi/2 - t/2, a/2 + t/2 - pi/2] ]

repNW6 = {t:3*pi/8, a:3*pi/2} # Replacement values in range

# Define conditions for model
condNW6 = [a >= pi, t <= pi/2, 2*pi - 2*t <= a, a <= 2*pi - t]

# Calculate model, run checks, write output.
pNW6 = calcModel(mNW6)
allChecks('pNW6')
parseLaTeX('pNW6')



# NW7 animal: a>pi.  Sensor: t <= pi/2. Condition: a <= 2pi - 2t  #


mNW7 = [ [2*r*sin(t/2)*sin(x2), x2, pi/2 - t/2, pi/2],
         [r*sin(x3),            x3, t,          pi/2],
         [r,                    x4, 0,          t   ],
         [r*sin(x3),            x3, pi - a/2,   pi/2] ]


repNW7 = {t:pi/9, a:10*pi/9} # Replacement values in range

# Define conditions for model
condNW7 = [t <= pi/2, a >= pi, a <= 2*pi - 2*t]

# Calculate model, run checks, write output.
pNW7 = calcModel(mNW7)
allChecks('pNW7')
parseLaTeX('pNW7')



# SE1 animal: a <= pi.  Sensor: t =2pi.            #

mSE1 = [ [ 2*r*sin(a/2),x1, pi/2, 3*pi/2       ],
         ]


repSE1 = {a:pi/4} # Replacement values in range

# Define conditions for model
condSE1 = [a <= pi]

# Calculate model, run checks, write output.
pSE1 = calcModel(mSE1)
allChecks('pSE1')
parseLaTeX('pSE1')




# SE2 animal: a <= pi.  Sensor: t > pi. Condition: a > 2pi - t, a > 4pi - 2t    #

mSE2 = [ [ 2*r*sin(a/2),                    x1, pi/2,                t/2 + pi/2 - a/2       ],
         [ r*sin(a/2) + r*cos(x1 - t/2),    x1, t/2 + pi/2 - a/2,   5*pi/2 - a/2 - t/2 ],
         [ 2*r*sin(a/2),                    x1, 5*pi/2 - a/2 - t/2, 3*pi/2]  ]


repSE2 = {t:19*pi/10, a:pi/2} # Replacement values in range

# Define conditions for model
condSE2 = [a <= pi, t >= pi,  a >= 4*pi - 2*t]

# Calculate model, run checks, write output.
pSE2 = calcModel(mSE2)
allChecks('pSE2')
parseLaTeX('pSE2')


# SE3 animal: a <= pi.  Sensor: t > pi. Condition: 2pi - t < a < 4pi - 2t #

mSE3 = [ [ 2*r*sin(a/2),                    x1, pi/2,                t/2 + pi/2 - a/2  ],
         [ r*sin(a/2) + r*cos(x1 - t/2),    x1, t/2 + pi/2 - a/2,   t/2 + pi/2        ],
         [ r*sin(a/2),                      x1, t/2 + pi/2,          5*pi/2 - a/2 - t/2],
         [ 2*r*sin(a/2),                    x1, 5*pi/2 - a/2 - t/2, 3*pi/2            ] ]

repSE3 = {t:3*pi/2 + 0.1, a:pi/2} # Replacement values in range

# Define conditions for model
condSE3 = [a <= pi, t >= pi,  a >= 2*pi - t, a <= 4*pi - 2*t]

# Calculate model, run checks, write output.
pSE3 = calcModel(mSE3)
```

```
allChecks('pSE3')
parseLaTeX('pSE3')


# SE4 animal: a <= pi.  Sensor: t > pi. Condition: a <= 4*pi - 2*t and a < 2*pi - t #


mSE4 = [ [ 2*r*sin(a/2),                      x1, pi/2,            t/2 + pi/2 - a/2  ],
         [ r*sin(a/2) + r*cos(x1 - t/2),      x1, t/2 + pi/2 - a/2, t/2 + pi/2       ],
         [ r*sin(a/2),                        x1, t/2 + pi/2,      t/2 + pi/2 + a/2  ] ]


repSE4 = {t:3*pi/2, a:pi/3} # Replacement values in range


# Define conditions for model
condSE4 = [a <= pi, t >= pi/2, a <= 4*pi - 2*t , a <= 2*pi - t]

# Calculate model, run checks, write output.
pSE4 = calcModel(mSE4)
allChecks('pSE4')
parseLaTeX('pSE4')


# SW1 animal: a <= pi.  Sensor: pi/2 <= t <= pi. Condition: a >= t and a/2 >= t - pi/2 #

mSW1 =  [ [2*r*sin(t/2)*sin(x2),              x2, pi/2 - a/2 + t/2, pi/2             ],
          [r*sin(a/2) - r*cos(x2 + t/2),      x2, t/2,              pi/2 - a/2 + t/2],
          [r*sin(a/2) - r*cos(x4 - t),        x4, 0,                t - pi/2         ],
          [r*sin(a/2),                        x4, t-pi/2,           t - pi/2 + a/2  ] ]


repSW1 = {t:5*pi/8, a:6*pi/8} # Replacement values in range

# Define conditions for model
condSW1 = [a <= pi, pi/2 <= t, t <= pi, a >= t, a/2 >= t - pi/2]

# Calculate model, run checks, write output.
pSW1 = calcModel(mSW1)
allChecks('pSW1')
parseLaTeX('pSW1')


# SW2 animal: a <= pi.  Sensor: pi/2 <= t <= pi. Condition: a <= t and a/2 >= t- pi/2 #

mSW2 =  [ [2*r*sin(a/2),                x2, pi/2 + a/2 - t/2, pi/2             ],
          [r*sin(a/2) - r*cos(x2 + t/2), x2, t/2,             pi/2 + a/2 - t/2],
          [r*sin(a/2) - r*cos(x4 - t),  x4, 0*t,              t - pi/2        ],
          [r*sin(a/2),                  x4, t - pi/2,         t - pi/2 + a/2 ] ]


repSW2 = {t:7*pi/8, a:7*pi/8-0.1} # Replacement values in range

# Define conditions for model
condSW2 = [a <= pi, pi/2 <= t, t <= pi, a/2 <= t/2, a/2 >= t - pi/2]

# Calculate model, run checks, write output.
pSW2 = calcModel(mSW2)
allChecks('pSW2')
parseLaTeX('pSW2')



# SW3 animal: a <= pi.  Sensor: pi/2 <= t <= pi. Condition: a <= t and a/2 <= t- pi/2 #

mSW3 =  [ [2*r*sin(a/2),                      x2, t/2,             pi/2             ],
          [2*r*sin(a/2),                      x4, 0,               t - pi/2 - a/2 ],
          [r*sin(a/2) - r*cos(x4 - t),        x4, t - pi/2 - a/2, t - pi/2         ],
          [r*sin(a/2),                        x4, t - pi/2,        t - pi/2 + a/2 ] ]


repSW3 = {t:7*pi/8, a:2*pi/8} # Replacement values in range

# Define conditions for model
condSW3 = [a <= pi, pi/2 <= t, t <= pi, a/2 <= t/2, a/2 <= t - pi/2]

# Calculate model, run checks, write output.
pSW3 = calcModel(mSW3)
allChecks('pSW3')
parseLaTeX('pSW3')


# SW4 animal: a <= pi.  Sensor: t <= pi/2. Condition: a > pi - 2t &  a <= t      #

mSW4 = [ [2*r*sin(a/2),                 x2, pi/2 - t/2 + a/2, pi/2             ],
         [r*sin(a/2) - r*cos(x2 + t/2), x2, pi/2 - t/2,       pi/2 - t/2 + a/2],
         [r*sin(a/2),                   x3, t,                pi/2             ],
```

```
          [r*sin(a/2),                    x4, 0,                a/2 + t - pi/2  ] ]

repSW4 = {t:pi/2-0.1, a:pi/4} # Replacement values in range

# Define conditions for model
condSW4 = [a <= pi,  t <= pi/2,  a >= pi - 2*t,  a <= t]

# Calculate model, run checks, write output.
pSW4 = calcModel(mSW4)
allChecks('pSW4')
parseLaTeX('pSW4')


# SW5 animal: a <= pi.  Sensor: t <= pi/2. Condition: a > pi - 2t &  t <= a <= 2t    #

mSW5 = [ [2*r*sin(t/2)*sin(x2),          x2, pi/2 + t/2 - a/2, pi/2             ],
          [r*sin(a/2) - r*cos(x2 + t/2), x2, pi/2 - t/2,       pi/2 + t/2 - a/2],
          [r*sin(a/2),                    x3, t,                pi/2         ],
          [r*sin(a/2),                    x4, 0,                a/2 + t -pi/2   ] ]


repSW5 = {t:pi/2-0.1, a:pi/2} # Replacement values in range

# define conditions for model
condSW5 = [a <= pi,  t <= pi/2,  a >= pi - 2*t,  t <= a, a <= 2*t]


# Calculate model, run checks, write output.
pSW5 = calcModel(mSW5)
allChecks('pSW5')
parseLaTeX('pSW5')


# SW6 animal: a <= pi.  Sensor: t <= pi/2. Condition: a > pi - 2t &  a > 2t      #

mSW6 = [ [2*r*sin(t/2)*sin(x2), x2, pi/2 - t/2, pi/2              ],
          [r*sin(x3),            x3, t,          a/2               ],
          [r*sin(a/2),           x3, a/2,        pi/2              ],
          [r*sin(a/2),           x4, 0,          a/2 + t -pi/2    ] ]


repSW6 = {t:pi/4, a:3*pi/4} # Replacement values in range


# Define conditions for model
condSW6 = [a <= pi,  t <= pi/2,  a >= pi - 2*t,  a > 2*t]

# Calculate model, run checks, write output.
pSW6 = calcModel(mSW6)
allChecks('pSW6')
parseLaTeX('pSW6')


# SW7 animal: a <= pi.  Sensor: t <= pi/2. Condition: a <= pi - 2t & a <= t     #

mSW7 = [ [2*r*sin(a/2),                   x2, pi/2 - t/2 + a/2, pi/2             ],
          [r*sin(a/2) - r*cos(x2 + t/2), x2, pi/2 - t/2,       pi/2 - t/2 + a/2],
          [r*sin(a/2),                    x3, t,                t + a/2         ] ]


repSW7 = {t:2*pi/8, a:pi/8} # Replacement values in range

# Define conditions for model
condSW7 = [a <= pi, t <= pi/2, a <= pi - 2*t, a <= t]

# Calculate model, run checks, write output.
pSW7 = calcModel(mSW7)
allChecks('pSW7')
parseLaTeX('pSW7')


# SW8 animal: a <= pi.  Sensor: t <= pi/2. Condition: a <= pi - 2t & t <= a <= 2t   #

mSW8 = [ [2*r*sin(t/2)*sin(x2),          x2, pi/2 + t/2 - a/2, pi/2             ],
          [r*sin(a/2) - r*cos(x2 + t/2), x2, pi/2 - t/2,       pi/2 + t/2 - a/2],
          [r*sin(a/2),                    x3, t,                t + a/2         ] ]

repSW8 = {t:2*pi/8, a:pi/2-0.1} # Replacement values in range

# Define conditions for model
condSW8 = [a <= pi, t <= pi/2, a <= pi - 2*t, t <= a, a <= 2*t]

# Calculate model, run checks, write output.
pSW8 = calcModel(mSW8)
allChecks('pSW8')
parseLaTeX('pSW8')

```

```
# SW9 animal: a <= pi.  Sensor: t <= pi/2. Condition: a <= pi - 2t &  2t <= a      #

mSW9 = [ [2*r*sin(t/2)*sin(x2), x2, pi/2 - t/2, pi/2    ],
         [r*sin(x3),            x3, t,          a/2     ],
         [r*sin(a/2),           x3, a/2,        t + a/2 ] ]


repSW9 = {t:1*pi/8, a:pi/2} # Replacement values in range

# Define conditions for model
condSW9 = [a <= pi,  t <= pi/2,  a <= pi - 2*t,  2*t <= a]

# Calculate model, run checks, write output.
pSW9 = calcModel(mSW9)
allChecks(’pSW9’)
parseLaTeX(’pSW9’)


####################
## Run tests      ###
####################

# create gas model object
gas = 2*r


# for each model run through every adjacent model.
# Contains duplicates but better for avoiding missed comparisons.
# Also contains replacement t->a and a->t just in case.


allComps = [
[’gas’, ’pNE1’, {t:2*pi}], [’gas’, ’pSE1’, {a:pi}],

[’pNE1’, ’gas’, {t:2*pi}], [’pNE1’, ’pNW1’, {t:pi}],
[’pNE1’, ’pNE2’,{a:3*pi-t}], [’pNE1’, ’pNE2’,{t:3*pi-a}],

[’pNE2’, ’pNE1’,{a:3*pi-t}], [’pNE2’, ’pNE1’,{t:3*pi-a}],
[’pNE2’, ’pNE3’,{a:4*pi-2*t}], [’pNE2’, ’pNE3’,{t:2*pi-a/2}],
[’pNE2’, ’pSE2’,{a:pi}],

[’pNE3’, ’pNE2’,{a:4*pi-2*t}], [’pNE3’, ’pNE2’,{t:2*pi-a/2}],
[’pNE3’, ’pSE3’,{a:pi}], [’pNE3’, ’pNW2’,{t:pi}],

[’pNW1’,’pNE1’, {t:pi}], [’pNW1’,’pNW2’,{a:2*pi}],

[’pNW2’,’pNE3’,{t:pi}], [’pNW2’,’pNW3’,{a:3*pi-2*t}],
[’pNW2’,’pNW3’,{t:3*pi/2-a/2}], [’pNW2’,’pNW1’,{a:2*pi}],

[’pNW3’,’pNW5’,{t:pi/2}], [’pNW3’,’pNW4’,{a:2*pi-t}],
[’pNW3’,’pNW4’,{t:2*pi-a}], [’pNW3’,’pNW2’,{a:3*pi-2*t}],
[’pNW3’,’pNW2’,{t:3*pi/2-a/2}],

[’pNW4’,’pNW6’,{t:pi/2}], [’pNW4’,’pNW3’,{t:2*pi-a}],
[’pNW4’,’pNW3’,{a:2*pi-t}], [’pNW4’,’pSW1’,{a:pi}],

[’pREM’,’pNW1’, {t:pi/2}], [’pREM’,’pNW5’,{a:2*pi}],

[’pNW5’,’pREM’,{a:2*pi}], [’pNW5’,’pNW6’,{a:2*pi-t}],
[’pNW5’,’pNW6’,{t:2*pi-a}], [’pNW5’,’pNW3’,{t:pi/2}],

[’pNW6’,’pNW5’,{a:2*pi-t}], [’pNW6’,’pNW5’,{t:2*pi-a}],
[’pNW6’,’pNW7’,{t:pi-a/2}], [’pNW6’,’pNW7’,{a:2*pi-2*t}],
[’pNW5’,’pNW4’,{t:pi/2}],

[’pNW7’,’pNW6’,{t:2*pi-2*a}], [’pNW7’,’pNW6’,{a:2*pi-2*t}],
[’pNW7’,’pSW6’,{a:pi}],

[’pSE1’,’pSE2’,{t:2*pi}], [’pSE1’,’gas’,{a:pi}],

[’pSE2’,’pSE3’,{t:2*pi-a/2}], [’pSE2’,’pSE3’,{a:4*pi-2*t}],
[’pSE2’,’pSE1’,{t:2*pi}], [’pSE2’,’pNE2’,{a:pi}],

[’pSE3’,’pSE2’,{a:4*pi-2*t}], [’pSE3’,’pSE2’,{t:2*pi-a/2}],
[’pSE3’,’pSE4’,{a:2*pi-t}], [’pSE3’,’pSE4’,{t:2*pi-a}],
[’pSE3’,’pNE3’,{a:pi}],

[’pSE4’,’pSE3’,{t:2*pi-a}], [’pSE4’,’pSE3’,{a:2*pi-t}],
[’pSE4’,’pSW3’,{t:pi}],

[’pSW1’,’pSW5’,{t:pi/2}], [’pSW1’,’pSW2’,{a:t}],
[’pSW1’,’pSW2’,{t:a}], [’pSW1’,’pNW4’,{a:pi}],

[’pSW2’,’pSW1’,{a:t}], [’pSW2’,’pSW1’,{t:a}],
[’pSW2’,’pSW4’,{t:pi/2}], [’pSW2’,’pSW3’,{a:2*t-pi}],
[’pSW2’,’pSW3’,{t:a/2+pi/2}],
```

```
['pSW3','pSW2',{t:a/2+pi/2}], ['pSW3','pSW2',{a:2*t-pi}],
['pSW3','pSE4',{t:pi}],


['pSW4','pSW7',{a:pi-2*t}], ['pSW4','pSW7',{t:pi/2-a/2}],
['pSW4','pSW5',{t:a}], ['pSW4','pSW5',{a:t}],
['pSW4','pSW2',{t:pi/2}],

['pSW5','pSW4',{t:a}], ['pSW5','pSW4',{a:t}],
['pSW5','pSW8',{t:pi/2-a/2}], ['pSW5','pSW8',{a:pi-2*t}],
['pSW5','pSW6',{a:2*t}], ['pSW5','pSW6',{t:a/2}],
['pSW5','pSW1',{t:pi/2}],

['pSW6','pSW9',{t:pi/2-a/2}], ['pSW6','pSW9',{a:pi-2*t}],
['pSW6','pSW5',{a:2*t}], ['pSW6','pSW5',{t:a/2}],
['pSW6','pNW7',{a:pi}],


['pSW7','pSW8',{t:a}], ['pSW7','pSW8',{a:t}],
['pSW7','pSW4',{t:pi/2-a/2}], ['pSW7','pSW4',{a:pi-2*t}],

['pSW8','pSW7',{a:t}], ['pSW8','pSW7',{t:a}],
['pSW8','pSW9',{a:2*t}], ['pSW8','pSW9',{t:a/2}],
['pSW8','pSW5',{a:pi-2*t}], ['pSW8','pSW5',{t:pi/2-a/2}],

['pSW9','pSW8',{a:2*t}], ['pSW9','pSW8',{t:a/2}],
['pSW9','pSW6',{a:pi-2*t}], ['pSW9','pSW6',{t:pi/2-a/2}]
]


# List of regions that touch a=0. Should equal 0 when a=0.
zeroRegions = ['pSW9', 'pSW8', 'pSW7', 'pSW4', 'pSW2', 'pSW3', 'pSE4', 'pSE3',  'pSE1']


# Run through all the comparisons. Need simplify(). Even together() gives some false negatives.

checkFile = open('checksFile.tex','w')

checkFile.write('All checks evaluated.\nTim Lucas - ' + str(datetime.now()) + '\n')
for i in range(len(allComps)):
        if (eval(allComps[i][0]).subs(allComps[i][2]) - eval(allComps[i][1]).subs(allComps[i][2])).simplify() == 0:
                checkFile.write(str(i) + ': ' + allComps[i][0]+ ' and ' +allComps[i][1]+': OK\n')
        else:
                checkFile.write(str(i) + ': ' + allComps[i][0]+ ' and ' +allComps[i][1]+': Incorrect\n')

for i in range(len(zeroRegions)):
        if eval(zeroRegions[i]).subs({a:0}).simplify() == 0:
                checkFile.write(zeroRegions[i] + ' at a = 0: OK\n')
        else:
                checkFile.write(zeroRegions[i] + ' at a = 0: Incorrect\n')

# pSE2 is slightly different. Only one corner touches a=0, so need theta value as well. I'm not sure why this isn't
# A problem for some other regions.
if pSE2.subs({a:0, t:2*pi}) == 0:
        checkFile.write('pSE2 at a = 0, t = 2pi: OK\n')
else:
        checkFile.write('pSE2 at a = 0, t = 2pi: Incorrect\n')
checkFile.close()


# And print to terminal
#for i in range(len(allComps)):
#        if not (eval(allComps[i][0]).subs(allComps[i][2]) - eval(allComps[i][1]).subs(allComps[i][2])).simplify() == 0:
#                print allComps[i][0] + ' and ' + allComps[i][1]+': Incorrect\n'


#####################################################################
### Define a a function that calculates p bar answer.            ####
#####################################################################

def calcP(A, T, R):
  assert (A <= 2*pi and A >= 0), "a is out of bounds. Should be in 0<a<2*pi"
  assert (T <= 2*pi and T >= 0), "s is out of bounds. Should be in 0<s<2*pi"

  if A > pi:
    if A < 4*pi - 2* T:
      p = pNW7.subs({a:A,  t:T, r:R}).n()
    elif A <= 3*pi -  T:
                    p = pNE2.subs({a:A,  t:T, r:R}).n()
    else:
                    p = pNE1.subs({a:A,  t:T, r:R}).n()
  else:
    if A < 4*pi - 2* T:
```

```
                         p = pSE3.subs({a:A,  t:T, r:R}).n()
    else:
                         p = pSE2.subs({a:A,  t:T, r:R}).n()
        return p


##############################
## Apply to entire grid   ###
##############################

# How many values for each parameter
nParas = 100

# Make a vector for a and s. Make an empty nParas x nParas array.
# Calculated profile sizes will go in pArray
tVec = np.linspace(0, 2*pi, nParas)
aVec = np.linspace(0, 2*pi, nParas)
pArray = np.zeros((nParas,nParas))

# Calculate profile size for each combination of parameters
for i in range(nParas):
        for j in range(nParas):
                pArray[i][j] = calcP(aVec[i], tVec[j], 1)

# Turn the array upside down so origin is at bottom left.
pImage = np.flipud(pArray)

# Plot and save.
pl.imshow(pImage, interpolation='none', cmap=pl.get_cmap('Blues') )

# Show or save image.
# pl.show()
# pl.savefig('/imgs/profilesCalculated.png')



#############################
#### Output R function.  ###
#############################

# To reduce mistakes, output R function directly from python.
# However, the if statements, which correspond to the bounds of each model, are not automatic.

Rfunc = open('supplementaryRscript.R', 'w')

Rfunc.write("""
# S4
# Supplementary R script from
# A generalised random encounter model for estimating animal density with remote sensor data
# Tim C.D. Lucas, Elizabeth A. Moorcroft, Robin Freeman, Marcus J. Rowcliffe, Kate E. Jones
#
# calcDensity is the main function to calculate density.
# It takes parameters z, alpha, theta, r, animalSpeed, t
# z - The number of camera/acoustic counts or captures.
# alpha - Call width in radians.
# theta - Sensor width in radians.
# r - Sensor range in metres.
# animalSpeed - Average animal speed in metres per second.
# t - Length of survey in sensor seconds i.e. number of sensors x survey duration.
#
# calcAbundance calculates abundance rather than density and requires an extra parameter
# area - In metres squared. The size of the region being examined.


# Internal function to calculate profile width as described in the text
calcProfileWidth <- function(alpha, theta, r){
        if(alpha > 2*pi | alpha < 0)
            stop('alpha is out of bounds. alpha should be in interval 0<a<2*pi')
        if(theta > 2*pi | theta < 0)
            stop('theta is out of bounds. theta should be in interval 0<a<2*pi')

  if(alpha > pi){
          if(alpha < 4*pi - 2*theta){
""" +
'           p <- ' + str(pNW7) +
'\n                } else if(alpha <= 3*pi - theta){'
'\n                         p <- ' + str(pNE2) +
'\n                } else {'
'\n                         p <- ' + str(pNE1) +
'\n                }'
'\n        } else {'
'\n         if(alpha < 4*pi - 2*theta){'
'\n                         p <- ' + str(pSE3) +
'\n     } else {'
'\n                         p <- ' + str(pSE2) +
'\n                }'
'\n        }'
```

```
'\n        return(p)'
'\n}' +
"""
# Calculate a population density. See above for units etc.
calcDensity <- function(z, alpha, theta, r, animalSpeed, t){
        # Check the parameters are suitable.
        if(z <= 0 | !is.numeric(z)) stop('Counts, z, must be a positive number.')
        if(animalSpeed <= 0 | !is.numeric(animalSpeed)) stop('animalSpeed must be a positive number.')
        if(t <= 0 | !is.numeric(t)) stop('Time, t, must be a positive number.')

        # Calculate profile width, then density.
        p <- calcProfileWidth(alpha, theta, r)
        D <- z/{animalSpeed*t*p}
        return(D)
}

# Calculate abundance rather than density.
calcAbundance <- function(z, alpha, theta, r, animalSpeed, t, area){
        if(area <= 0 | !is.numeric(area)) stop('Area must be a positive number')
        D <- calcDensity(z, alpha, theta, r, animalSpeed, t)
        A <- D*area
        return(A)
}
"""
)

Rfunc.close()
```

lucas_et_al_S3.py

## S4. Supplementary Script: R implementation of models

This is an implementation of the models derived in the paper in R (**?**). Once given the parameters $\theta$ and $\alpha$ it automatically selects the correct model to apply.

The script can also be found in the plain text file `lucas_et_al_S4.R`. It will also be hosted and given a DOI on Github at the time of publication (`https://github.com/timcdlucas/lucasMoorcroftManuscript/tree/postPeerReview`).

```

# S4
# Supplementary R script from
# A generalised random encounter model for estimating animal density with remote sensor data
# Tim C.D. Lucas, Elizabeth A. Moorcroft, Robin Freeman, Marcus J. Rowcliffe, Kate E. Jones
#
# calcDensity is the main function to calculate density.
# It takes parameters z, alpha, theta, r, animalSpeed, t
# z - The number of camera/acoustic counts or captures.
# alpha - Call width in radians.
# theta - Sensor width in radians.
# r - Sensor range in metres.
# animalSpeed - Average animal speed in metres per second.
# t - Length of survey in sensor seconds i.e. number of sensors x survey duration.
#
# calcAbundance calculates abundance rather than density and requires an extra parameter
# area - In metres squared. The size of the region being examined.


# Internal function to calculate profile width as described in the text
calcProfileWidth <- function(alpha, theta, r){
    if(alpha > 2*pi | alpha < 0)
  stop(’alpha is out of bounds. alpha should be in interval 0<a<2*pi’)
    if(theta > 2*pi | theta < 0)
  stop(’theta is out of bounds. theta should be in interval 0<a<2*pi’)

 if(alpha > pi){
     if(alpha < 4*pi - 2*theta){
      p <- r*(theta - cos(alpha/2) + 1)/pi
        } else if(alpha <= 3*pi - theta){
            p <- r*(theta - cos(alpha/2) + cos(alpha/2 + theta))/pi
        } else {
            p <- r*(theta + 2*sin(theta/2))/pi
        }
    } else {
     if(alpha < 4*pi - 2*theta){
            p <- r*(theta*sin(alpha/2) - cos(alpha/2) + 1)/pi
  } else {
            p <- r*(theta*sin(alpha/2) - cos(alpha/2) + cos(alpha/2 + theta))/pi
        }
    }
    return(p)
}
# Calculate a population density. See above for units etc.
calcDensity <- function(z, alpha, theta, r, animalSpeed, t){
    # Check the parameters are suitable.
    if(z <= 0 | !is.numeric(z)) stop(’Counts, z, must be a positive number.’)
    if(animalSpeed <= 0 | !is.numeric(animalSpeed)) stop(’animalSpeed must be a positive number.’)
    if(t <= 0 | !is.numeric(t)) stop(’Time, t, must be a positive number.’)

    # Calculate profile width, then density.
    p <- calcProfileWidth(alpha, theta, r)
    D <- z/{animalSpeed*t*p}
    return(D)
}

# Calculate abundance rather than density.
calcAbundance <- function(z, alpha, theta, r, animalSpeed, t, area){
    if(area <= 0 | !is.numeric(area)) stop(’Area must be a positive number’)
    D <- calcDensity(z, alpha, theta, r, animalSpeed, t)
    A <- D*area
    return(A)
}
```

lucas_et_al_S4.R

## S5. Supplementary Information: Simulation model results of the gREM precision

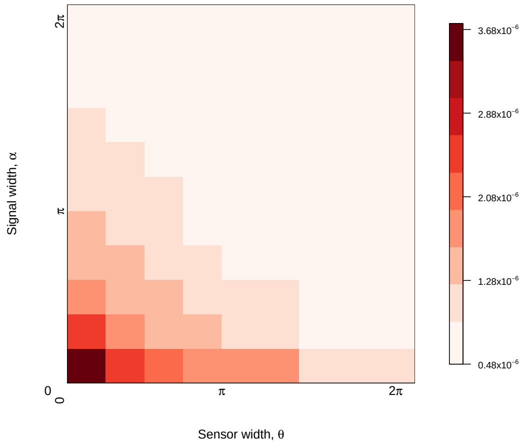


Figure S5.1. Simulation model results of the gREM precision given a range of sensor and signal widths, shown by the standard deviation of the error between the estimated and true densities. Standard deviations are shown from deep red to pink, representing high to low values between $0.483 \times 10^{-6}$ to $3.74 \times 10^{-6}$.

## S6. Supplementary Information: Impact of parameter error

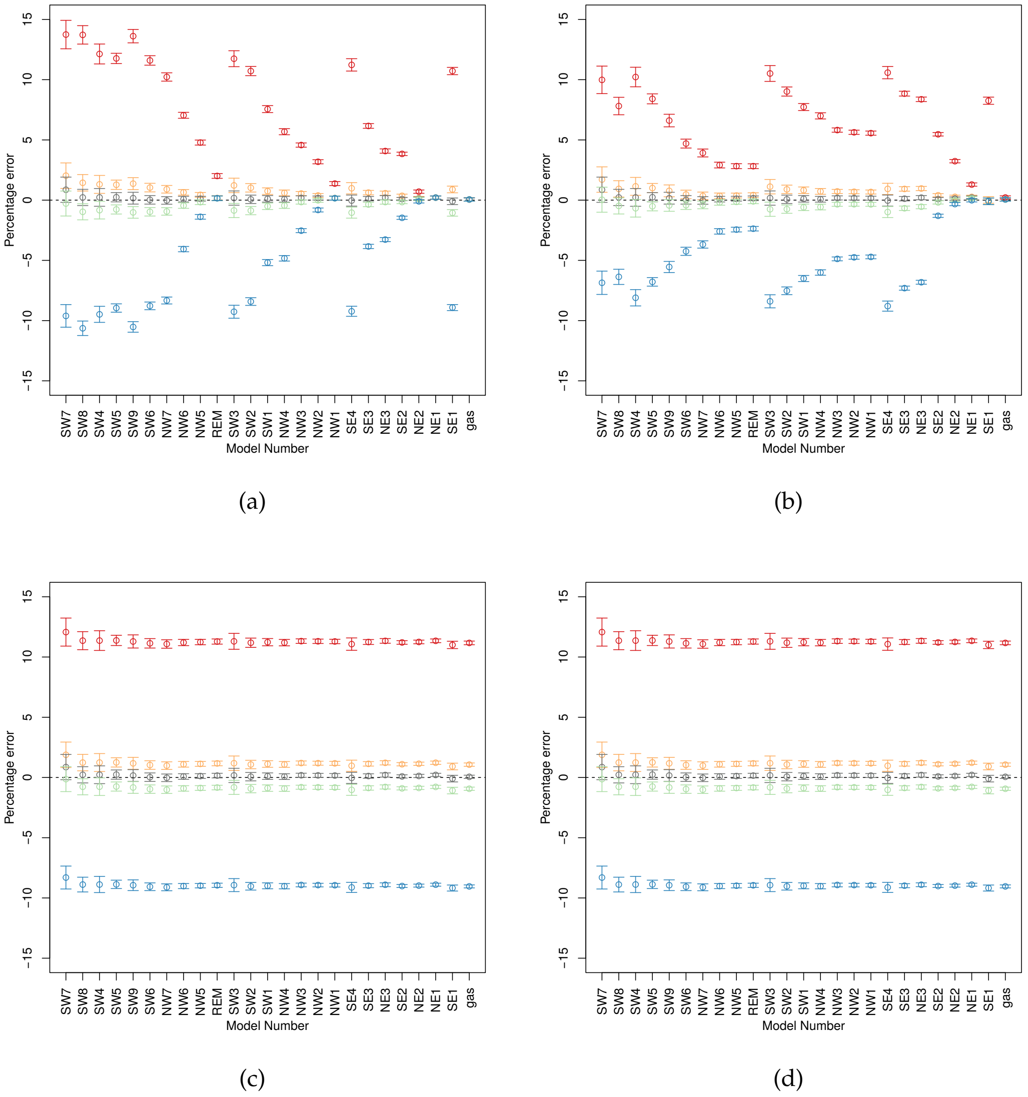


Figure S6.1. Model sensitivity (for all gREM submodels) to error in estimates of a) signal width $\alpha$, b) sensor width $\theta$, c) detection distance $r$ and d) animal movement speed $v$. Estimates are -10% (red), -1% (orange), 0% (grey), +1% (green) and +10% (blue) of the true parameter value. The black dashed line indicates zero error in density estimates. The error bars 95% confidence intervals across all simulations.
